# Synaptosome microRNAs regulate synapse functions in Alzheimer’s disease

**DOI:** 10.1038/s41525-022-00319-8

**Published:** 2022-08-08

**Authors:** Subodh Kumar, Erika Orlov, Prashanth Gowda, Chhanda Bose, Russell H. Swerdlow, Debomoy K. Lahiri, P. Hemachandra Reddy

**Affiliations:** 1 Center of Emphasis in Neuroscience, Department of Molecular and Translational Medicine, Texas Tech University Health Sciences Center, 5001 El Paso Drive, El Paso, TX 79905 USA; 2 Internal Medicine Department, Texas Tech University Health Sciences Center, 3601 4th Street STOP 9410, Lubbock, TX 79430 USA; 3 Paul L. Foster School of Medicine, Texas Tech University Health Sciences Center, 5001 El Paso Drive, El Paso, TX 79905, USA; 4 Department of Neurology, the University of Kansas Medical Center, University of Kansas Alzheimer’s Disease Research Center, Fairway, KS 66205 USA; 5 Laboratory of Molecular Neurogenetics’ Departments of Psychiatry and Medical & Molecular Genetics, Indiana University School of Medicine’ Indiana Alzheimer’s Disease Research Center, Stark Neuroscience Research Institute, Indianapolis, IN 46202 USA; 6 Department of Pharmacology & Neuroscience, Texas Tech University Health Sciences Center, 3601 4th Street STOP 9410, Lubbock, TX 79430 USA; 7 Department of Neurology, Texas Tech University Health Sciences Center, 3601 4th Street STOP 9410, Lubbock, TX 79430 USA; 8 Department of Public Health, Texas Tech University Health Sciences Center, 3601 4th Street STOP 9410, Lubbock, TX 79430 USA

**Keywords:** Molecular medicine, Genetics

## Abstract

MicroRNAs (miRNAs) are found in nerve terminals, synaptic vesicles, and synaptosomes, but it is unclear whether synaptic and cytosolic miRNA populations differ in Alzheimer’s disease (AD) or if synaptosomal miRNAs affect AD synapse activity. To address these questions, we generated synaptosomes and cytosolic fractions from postmortem brains of AD and unaffected control (UC) samples and analyzed them using a global Affymetrix miRNAs microarray platform. A group of miRNAs significantly differed (*P* < 0.0001) with high fold changes variance (+/− >200-fold) in their expressions in different comparisons: (1) UC synaptosome vs UC cytosol, (2) AD synaptosomes vs AD cytosol, (3) AD cytosol vs UC cytosol, and (4) AD synaptosomes vs UC synaptosomes. MiRNAs data analysis revealed that some potential miRNAs were consistently different across sample groups. These differentially expressed miRNAs were further validated using AD postmortem brains, brains of APP transgenic (Tg2576), Tau transgenic (P301L), and wild-type mice. The miR-501-3p, miR-502-3p, and miR-877-5p were identified as potential synaptosomal miRNAs upregulated with disease progression based on AD Braak stages. Gene Ontology Enrichment and Ingenuity Pathway Analysis of synaptosomal miRNAs showed the involvement of miRNAs in nervous system development, cell junction organization, synapse assembly formation, and function of GABAergic synapse. This is the first description of synaptic versus cytosolic miRNAs in AD and their significance in synapse function.

## Introduction

Alzheimer’s disease (AD) progresses with synaptic failure caused by amyloid beta (Aβ) and phosphorylated tau (p-tau) toxicities at synapses. In aged individuals, the number of AD cases are increasing gradually, and by mid‐century, the number of Americans age, 65 and older with Alzheimer’s dementia may grow to 13.8 million^1^. This represents a steep increase from the estimated 5.8 million Americans age, 65 and older who have Alzheimer’s dementia today.

Synaptic dysfunction or poor pre-synaptic and postsynaptic activities leads to the synaptic degeneration and neuron death in AD^2–4^. It is well known that synapse loss and dysfunction are the main physiological and pathological hallmarks of AD^5–8^.

Synapses are the key components for healthy brain functioning. Synapse integrity (number, structure, and functions) are crucial for balanced neurotransmission and to maintain healthy synaptic and cognitive functions of the brain. Synapse components can be extracted from postmortem brains in an intact form referred as “synaptosome or synapto-neurosomes”. Synaptosomes are the best neural cell component to study the synapse dysfunction in multiple neurodegenerative diseases, particularly in AD, where the synaptosome structure and functions are altered due to Aβ and p-tau accumulations^9^. During early AD progression, synapses are the first targets that are hit by Aβ and p-tau toxicities^10–12^. Multiple synaptic events are disturbed in AD, such as axonal transport, synapse mitochondrial function, synaptic vesicle trafficking, release and cycling, alteration of Ca^++^ influx, neurotransmitter release, impaired receptors, inflammation, and synaptotoxicity^9,13–19^.

MicroRNAs (miRNAs) are present throughout cells^9^. Some miRNAs are localized to subcellular compartments, including the rough endoplasmic reticulum, processing (P)-bodies, stress granules the trans-Golgi network, early/late endosomes, multivesicular bodies, lysosomes and mitochondria^9,20^. Several studies identified the presence of miRNAs at the synapse and in synaptosomal fractions and determined their important roles in the regulation of local protein synthesis^21–24^. Even synaptic vesicles extracted from mouse central nervous system that contain several small RNAs, transfer-RNAs and miRNAs^23^. In addition, miRNAs were found to be abundantly expressed within synaptoneurosomes isolated from prion-infected forebrain^24^.

Since the 1980s, researchers began using synaptosomes prepared from postmortem brains to study AD-associated deficits in neurotransmission, including dysfunction of excitatory synapse acetylcholine, glutamate or aspartate, and inhibitory synapse glycine or GABA (gamma-aminobutyric acid) systems^25–27^. A decrease in GABAergic synapse activity and inhibitory interneurons could contribute to AD progression and cognitive deficits in human and AD mouse models^28–31^. Synaptic disturbances at the excitatory and inhibitory synapse in the forebrain have been found to contribute the progression of AD and dementia^27^. Recent synaptosomal studies have revealed decreased levels of neprilysin in AD patients^32^. Neprilysin plays a key role in the clearance of Aβ.

Recently, it is well acknowledged that miRNAs exert widespread regulation over the translation and degradation of their target genes in the nervous system^33–35^. Increasing evidence suggests that quite a few specific miRNAs play important roles in various aspects of synaptic plasticity, including synaptic activity, synaptic development, synaptogenesis, synaptic morphology, synaptic remodeling, synaptic scaling, synaptic excitability, synaptic ATP production, and synaptic integrity^9,19,36–40^. More importantly, the miRNA-mediated regulation of synaptic plasticity is not only responsible for synapse development and function but is also involved in the pathophysiology of plasticity-related diseases, including AD^19,37,38^.

MiRNAs are the potential regulators of gene(s) and gene products and their therapeutic relevance have been explored in human diseases, including AD^41–47^. The role of miRNAs has been exposed in the regulation of synaptic activity in the case of AD^9^.

MiRNAs that enrich at the synapse directly regulate local protein synthesis involved in multiple synaptic functions and governing synaptic plasticity^21–24,48,49^. However, the role of synaptosome-specific miRNAs is not determined in the progression of AD. There are no published reports about synaptosome-specific miRNAs for AD thus far. Furthermore, it is unclear whether synaptosomal miRNAs are different from cytosolic miRNAs. Hence, this study classified synaptosomal versus cytosolic miRNAs and unfurled the possible molecular link between synaptosomal miRNAs and AD progression. Our study addressed four previously unknown important research questions: (1) Are miRNA(s) levels altered at the synaptosome in AD? (2) If so, are synapse miRNAs expressed differently in AD than in a healthy state? (3) Are synaptosomal miRNAs expressed differentially in the cytosol? and (4) What function do synaptosomal miRNAs play in synaptic activity and neurotransmission in AD? Overall, the focus of this study is to discover synaptosomal miRNAs and understand their positive and negative roles in AD progression.

## Results

### Synaptosomes preparations from postmortem brains

**Fig. 1:**
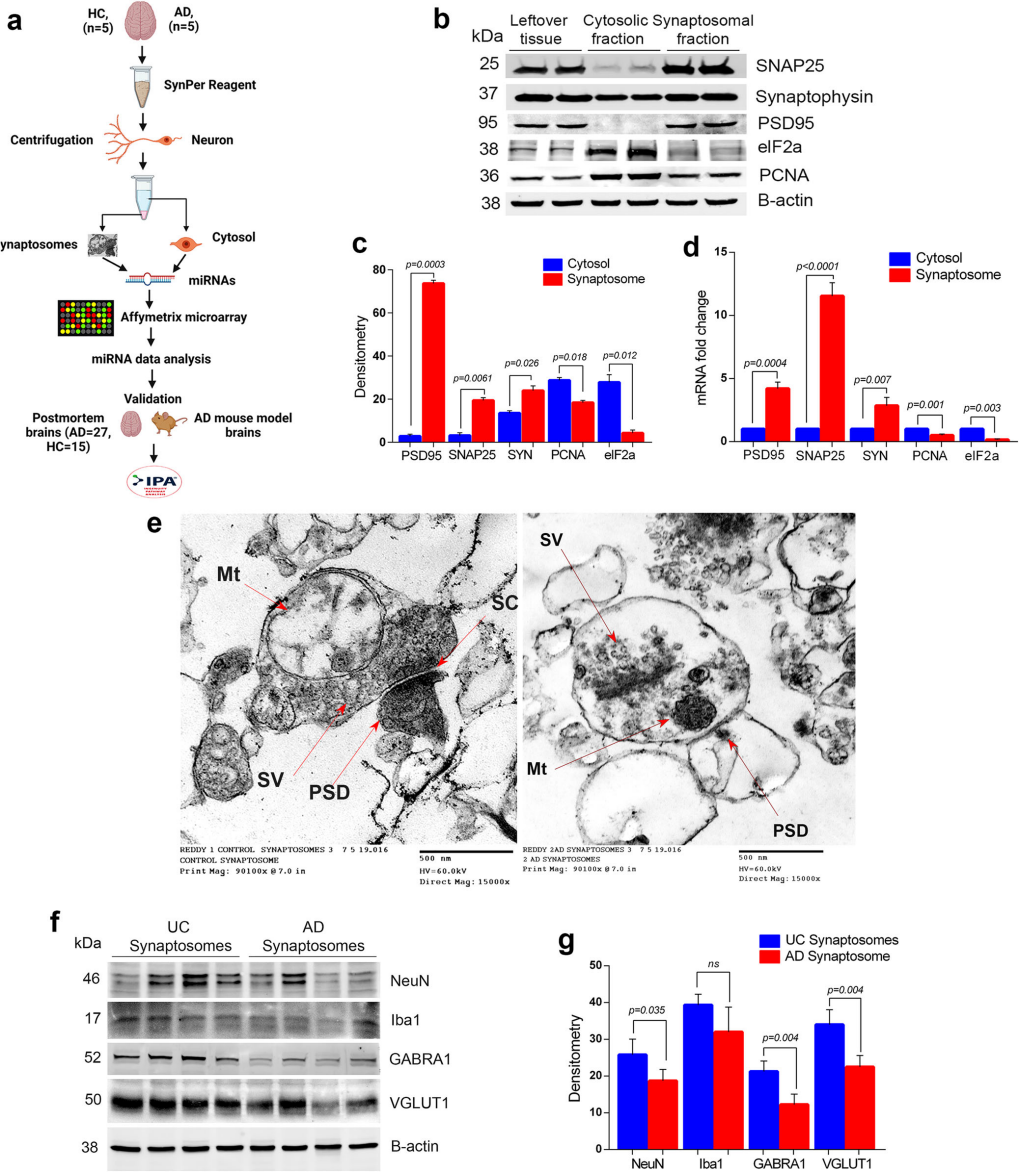
Extraction and characterization of synaptosomes. **a** Brief workflow of the current study. **b** Immunoblotting analysis of synaptic (SNAP25, synaptophysin and PSD95) and cytosolic (elF1a and PCNA) proteins in cytosolic fraction, synaptosomal fraction and leftover tissue debris of unaffected control postmortem brain tissues. **c** Densitometry analysis of synaptic and cytosolic proteins. Synaptic proteins levels (PSD95; *P* = 0.003), (SNAP25; *P* = 0.0061), (Synaptophysin; *P* = 0.026) were significantly higher in synaptosomes and cytosolic proteins (elF1a; *P* = 0.012) and (PCNA; *P* = 0.018) levels were significantly lower in synaptosomes relative to cytosol. **d** qRT-PCR analysis for mRNA fold change analysis of synaptic and cytosolic genes in cytosolic and synaptosomal fractions (*n* = 5). **e** TEM analysis of synapse assembly in synaptosomal fraction from unaffected control and AD patients’ postmortem brains (scale bar 500 nm magnification). Electron micrograph shows synapse components: Mt mitochondria, SV synaptic vesicles, PSD postsynaptic density, SC synaptic cleft. **f** Immunoblotting analysis of brain cells markers (Neuron-NeuN; Microglia-Iba1), excitatory synapse marker (VGLUT1) and inhibitory synapse marker (GABARA1) proteins in unaffected controls (*n* = 4) and AD (*n* = 4) synaptosomes. **g** Densitometry analysis of NeuN, Iba1, VGLUT1, and GABARA1 proteins in unaffected controls and AD synaptosomes. All blots are driven from the same experiment and were proceed parallelly (**b**, **f**). Values in the bar diagrams are mean ± SEM and error bars are equivalent throughout the figure (**c**, **d**, **g**).

Increased levels of APP (c-terminal fragment) and p-tau proteins were detected in the AD cases compared to UC samples, especially in the cytosolic fraction (Supplementary Fig. 1). Next, these samples were processed for synaptosome preparation and downstream applications (Fig. 1a). Figure 1b showed a representative immunoblot for SNAP25, synaptophysin, and PSD95 and cytosolic/nuclear proteins elF1a and PCNA. Densitometry analysis showed significantly increased levels of SNAP25, synaptophysin, and PSD95 in the synaptosome fraction and reduced levels in the cytosolic fraction (Fig. 1c). SNAP25 and PSD95 were completely absent from the cytosolic fraction, however, synaptophysin was detected in the cytosolic fraction, which was also as reported by other researchers^50^. On the other hand, elF1a and PCNA protein levels were higher in cytosol. qRT-PCR analysis also showed increased expression of SNAP25, synaptophysin, and PSD95 genes in the synaptosomes relative to the cytosol and reduced expressions of elF1a and PCNA in the synaptosomes fraction relative to the cytosol (Fig. 1d). These results confirm a precise separation of cytosolic and synaptosomes fractions.

Next, we processed the synaptosomes fraction from AD patients and UC for TEM analysis (Fig. 1e). The electron micrograph revealed the distinct synapse assembly and intact synaptosomes with all the components- mitochondria, synaptic vesicles, endosomes, postsynaptic density protein, and synaptic cleft. The mitochondrial structure and synaptic clefts were found to be distorted in AD postmortem brains and UC postmortem brains; however, mitochondrial distortion was more in AD cases. These results confirmed the purity and integrity of synapse and synaptosomes fraction.

Further, to confirm the brain cells specificity of synaptosomes, we checked the levels of cell type markers (NeuN-Neuron, Iba1-Microglia, GFAP-Astrocytes). We found significantly detectable levels of NeuN and Iba1 proteins (but not GFAP) in both UC and AD synaptosomes (Fig. 1f). NeuN level was found to be significantly reduced (*P* = 0.035) in AD synaptosomes relative to UC synaptosomes (Fig. 1g). We did not see any significant difference in Iba1 levels in AD vs UC synaptosome. These observations confirm the neuron specificity of synaptosomes.

We also characterized the synaptosomes as excitatory or inhibitory based on the levels of excitatory synapse marker Vesicular glutamate transporter 1 (VGLUT1) and inhibitory synapse markers Gamma-Aminobutyric Acid Type A Receptor Subunit Alpha1 (GABRA1). Immunoblots in Fig. 1f showed the levels of both markers in UC and AD synaptosomes. The levels of VGLUT1 (*P* = 0.004) and GABRA1 (*P* = 0.004) proteins were significantly reduced in AD synaptosomes relative to UC synaptosomes (Fig. 1g). These observations confirmed the presence of both types of synapses in synaptosomes fraction with their reduction in AD brains.

### MicroRNAs expression in UC synaptosomes vs UC cytosol

The miRNA microarray data of synaptosomal and cytosolic fractions were analyzed by Transcription analysis console v.4. A total of 43 mature miRNAs were found to be differentially distributed in UC synaptosomal fraction and UC cytosolic fraction (Supplementary Table 4). As shown in Supplementary Table 4, the 20 Homosapiens (hsa) miRNAs were highly expressed in the synaptosomes and low in the cytosol. These observations indicate that highly expressed miRNAs in synaptosomes have functional importance in synapse function. The 23 hsa-miRNAs (Supplementary Table 4) were highly expressed in the cytosol and showed reduced expression in the synaptosomes, strongly suggesting that these miRNAs have cytosolic relevance in the healthy state.

**Fig. 2:**
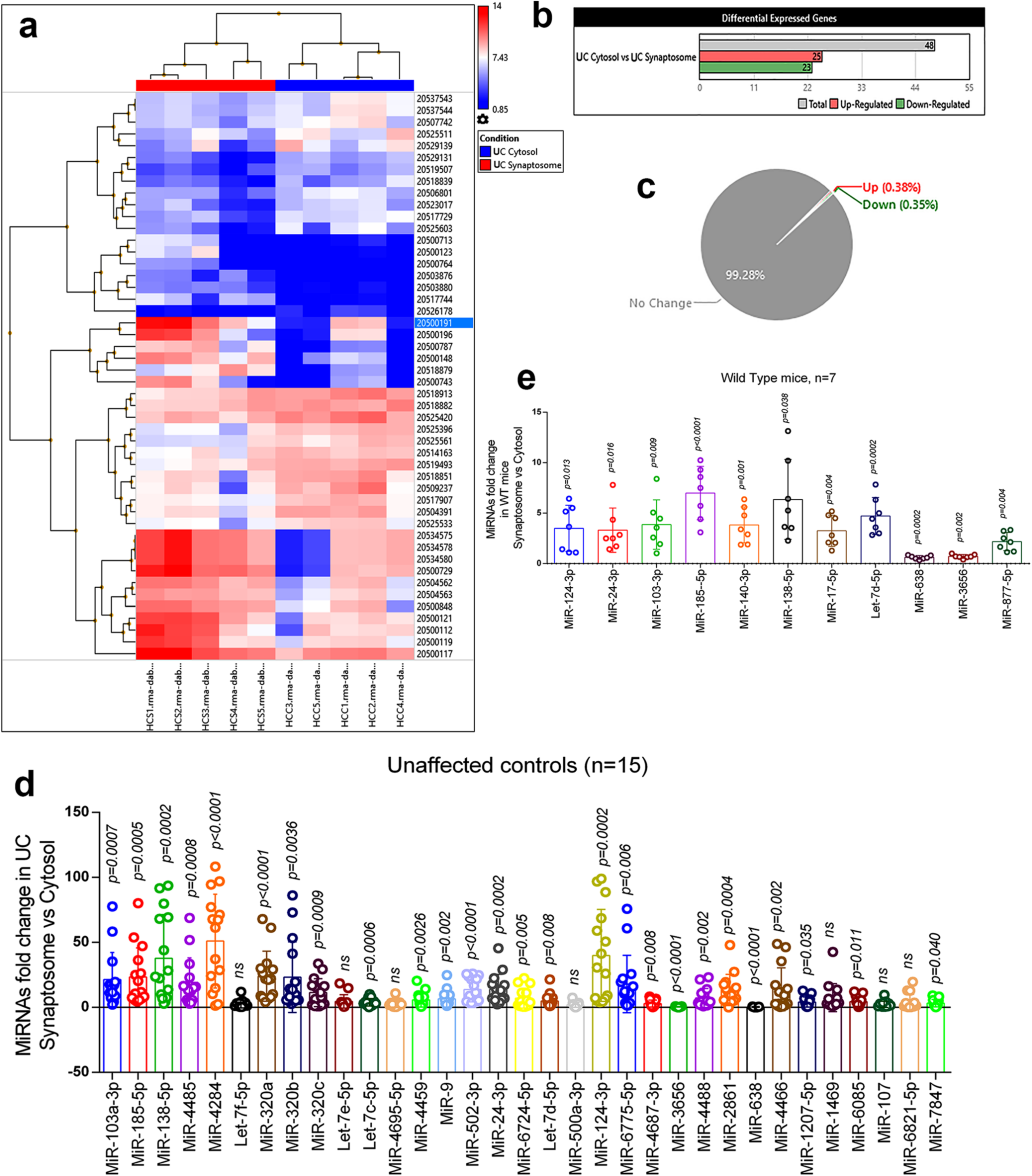
MiRNAs expression in synaptosome and cytosol in a healthy state. **a** Hierarchical clustering and heatmap of significantly deregulated miRNAs in the synaptosome and cytosol of unaffected controls. (red color intensity showed the miRNAs upregulation and blue color intensity showed the miRNAs downregulation). **b** Total number of miRNAs deregulated in cytosol vs synaptosome in unaffected controls. (grayscale bar—total number of miRNAs; red scale bar—upregulated miRNAs; green scale bar—downregulated miRNAs). **c** Pi diagram showed the total miRNAs pool distribution and percentage of miRNAs population changed in cytosol and synaptosome in unaffected controls. **d** qRT-PCR-based validation analysis of significantly deregulated miRNAs in unaffected controls (*n* = 15). MiRNAs expression was quantified in terms of fold changes in unaffected controls synaptosomes compared to the cytosol. Each circle dot represents one sample. **e** Validation analysis of significantly deregulated mmu-miRNAs in WT mice (*n* = 7). MiRNAs expression was quantified in synaptosome relative to the cytosol. Each circle dot represents one animal. Values in the bar diagrams are mean ± SEM and error bars are equivalent throughout the figure (**e**, **d**).

MiRNAs were characterized on several selection criteria—fold change, standard deviation, *P* values, expression priority, transcript ID, chromosome location, strand specificity, start and stop codon, targeted, and validated gene symbols (Supplementary Table 4). Figure 2a shows the hierarchical clustering and heatmap of significantly distributed miRNAs with their ID numbers. As a result, 25 miRNAs were upregulated, and 23 miRNAs were downregulated significantly (Fig. 2b). Gene-filter analysis of the total miRNAs pool shows that 99.28% of miRNA population did not show a significant difference in the cytosol vs synaptosome compartments. Only 0.38% population of miRNAs is upregulated, and 0.35% miRNA population is downregulated (Fig. 2c). The scattered plot shows the average log2 fold changes values of miRNAs with different distributions in cytosol vs synaptosomes (Supplementary Fig. 2a) and the volcano plot shows the *P* values (−log10) of significantly deregulated miRNAs (Supplementary Fig. 2b). The top candidate miRNAs were selected for validation analysis.

### Validation analysis of synaptosomal and cytosolic miRNAs in a healthy state

*(i) UC postmortem brains:* Validation analysis was performed on UC (*n* = 15) postmortem brains to distinguish synaptosomal and cytosolic miRNAs in the normal state. Out of the 43 deregulated miRNAs, only 33 miRNAs were successfully amplified by qRT-PCR using specific primers. The 18 miRNAs showed similar expression trends as obtained by Affymetrix data analysis. The remaining miRNAs did not concur with Affymetrix data. Overall, 24 miRNAs were significantly upregulated in the synaptosomes relative to the cytosol, and two miRNAs (miR-638 and miR-3656) were significantly downregulated in the synaptosomal fractions relative to the cytosolic fractions. Seven miRNAs did not show any significant changes (Fig. 2d).

*(ii) WT mice brains:* Further, we performed expression analysis of the above classified synaptosomal and cytosolic miRNAs in WT mice (*n* = 7). A total of 11 Mus musculus (mmu)-miRNAs were amplified, and out of them, nine were significantly upregulated and two were downregulated in WT mice synaptosome relative to the cytosol (Fig. 2e). The 11 miRNAs showed similar expression pattern as observed by primary screening and UC postmortem brain validation. Based on these observations, nine miRNAs were classified as synaptosomal miRNAs and two miRNAs as cytosolic miRNAs in the healthy state.

### MicroRNAs expression in AD synaptosomes vs AD cytosol

Next, we compared the microarray data for miRNAs expression changes in AD synaptosomal fractions vs AD cytosolic fractions. A total of 39 mature miRNAs were found to be differentially distributed in AD synaptosome vs AD cytosol comparison as shown in Supplementary Table 5, and 28 hsa-miRNAs were highly expressed in the synaptosomes and low in the cytosol. The 11 out 39 miRNAs were highly expressed in the cytosol and showed reduced expression in the synaptosomes. The differential distribution of these miRNAs in the AD synaptosomes and AD cytosol suggests their functional relevance in the diseased state.

**Fig. 3:**
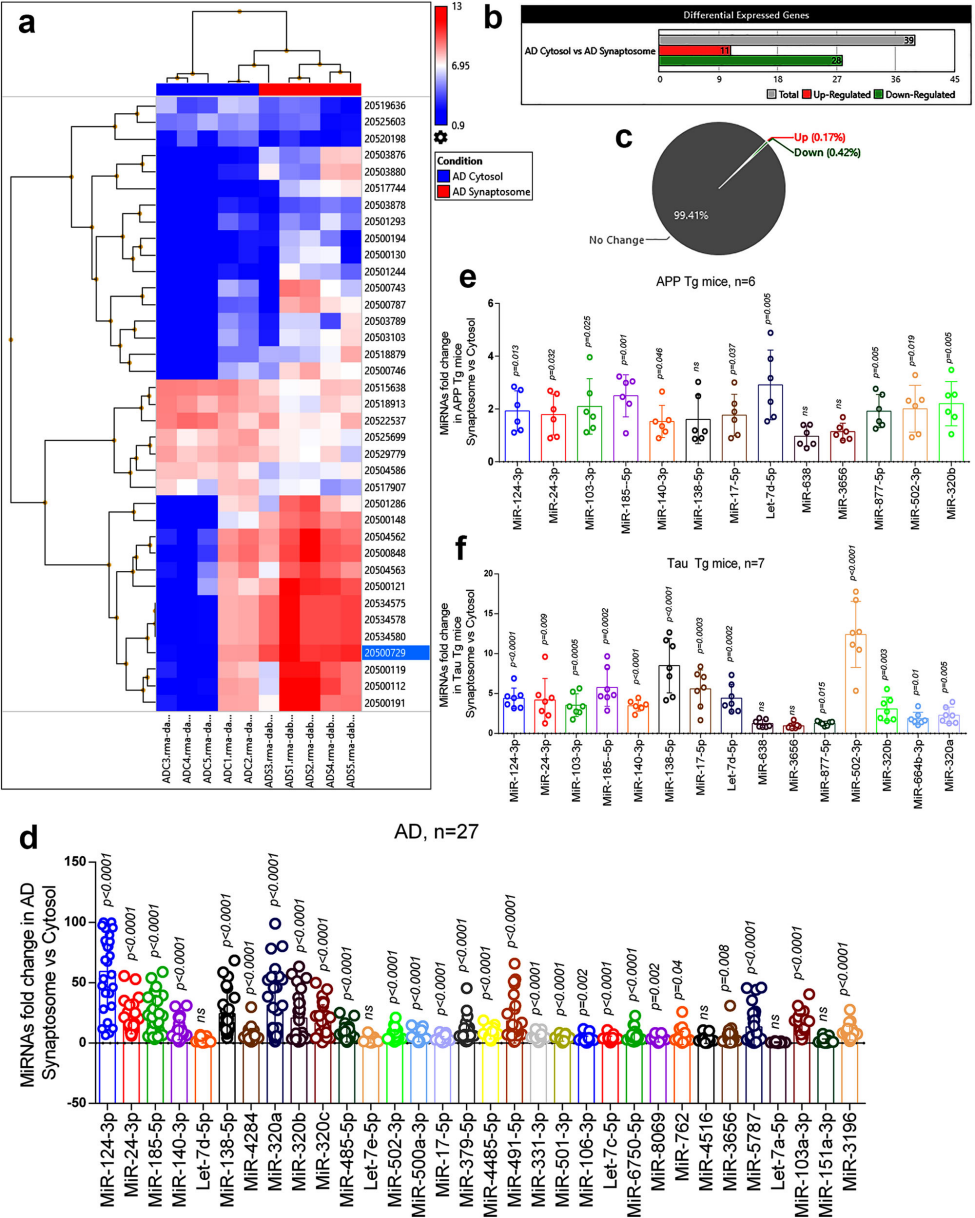
MiRNAs expression in synaptosome and cytosol in AD. **a** Hierarchical clustering and heatmap of significantly deregulated miRNAs in cytosol and synaptosome in AD samples. (red color intensity showed the miRNAs upregulation and blue color intensity showed the miRNAs downregulation) **b** Total numbers of miRNAs deregulated in cytosol and synaptosome in AD. (grayscale bar—total number of miRNAs; red scale bar—upregulated miRNAs; green scale bar—downregulated miRNAs). **c** Pi diagram showed the total miRNAs pool distribution and percentage of miRNA populations changed in cytosol and synaptosome. **d** qRT-PCR-based validation analysis of significantly deregulated miRNAs in AD samples (*n* = 27). MiRNAs expression was quantified in terms of fold changes in AD synaptosome compared to AD cytosol. Each circle dot represents one sample. **e** Validation analysis of significantly deregulated mmu-miRNAs in APP-Tg (*n* = 6) mice. MiRNAs expression was quantified in synaptosome relative to the cytosol. Each circle dot represents one animal. **f** Validation analysis of significantly deregulated mmu-miRNAs in Tau-Tg (*n* = 7) mice. MiRNAs expression was quantified in synaptosome relative to the cytosol. Values in the bar diagrams are mean ± SEM and error bars are equivalent throughout the figure (**d**–**f**).

Figure 3a shows hierarchical clustering and a heatmap of significantly distributed miRNAs with their ID numbers. The 11 miRNAs were upregulated in the cytosol and 28 miRNAs were downregulated in the cytosol significantly (Fig. 3b). Gene-filter analysis of the total miRNAs pool shows that 99.41% of miRNA population did not show a significant difference in the cytosol vs synaptosome compartment, only, 0.59% of populations showed variable expression levels. The 0.17% of miRNAs are upregulated, and 0.42% of miRNAs population is downregulated (Fig. 3c). The scattered plot shows the average log2 fold changes values of significantly deregulated miRNAs (Supplementary Fig. 2c) and the volcano plot shows the *P* values (−log10) of significantly deregulated miRNAs in AD synaptosome vs AD cytosol (Supplementary Fig. 2d). Based on the miRNA(s) expression pattern in unaffected controls and AD samples, 22 miRNAs (37.3%) were expressed only in UC samples and 21 miRNAs (35.6%) were expressed only in AD samples. However, 16 miRNAs (27.1%) were commonly expressed in both conditions (Supplementary Fig. 3).

### Validation analysis of synaptosomal and cytosolic miRNAs in AD state

*(i) AD postmortem brains:* The top candidate miRNAs were selected for validation analysis. Validation analyses were performed on 27 AD postmortem brains to distinguish synaptosomal and cytosolic miRNAs in the diseased state. Out of the 39 deregulated miRNAs, 32 miRNAs were amplified by using specific primers. The 22 miRNAs showed a similar expression trend as obtained by Affymetrix data analysis. The remaining miRNAs either showed opposite trend to Affymetrix data or did not change significantly. This could be due to large number of samples that were used for validation of initial Affymetrix analysis and possible pathological (Braak stages) differences of samples, may be likely reasons for inconsistent expression of miRNAs in two systems. Overall, 27 miRNAs were significantly upregulated in the synaptosomes relative to the cytosol and no miRNA showed any significant downregulation. The five miRNAs did not show any significant changes in the synaptosomes relative to the cytosol (Fig. 3d).

*(ii) APP-Tg mice:* Next, we did synaptosomal and cytosolic miRNAs validation using APP-Tg mice (*n* = 6). The 13 mmu-miRNAs showed similar expression pattern as observed by primary screening and AD postmortem brain validation. MiR-103-3p, miR-185-5p, miR-24-3p, miR-502-3p, miR-320b, let-7d-5p, miR-124-3p, miR-140-3p, miR-17-5p, and miR-877-5p showed significant upregulation in the synaptosomes, while miR-138-5p, miR-3656, and miR-638 did not show any significantly changes in their expression (Fig. 3e).

*(iii) Tau-Tg mice****:*** Further, we did synaptosomes and cytosolic miRNAs validation using Tau-Tg mice (n = 7). The 13 mmu-miRNAs showed similar expression pattern as observed by primary screening and AD postmortem brain validation. MiR-103-3p, miR-185-5p, miR-24-3p, miR-502-3p, miR-320b, let-7d-5p, miR-124-3p, miR-140-3p, miR-17-5p, miR-877-5p, miR-320a, and miR-664a-3p showed significant upregulation in the synaptosomes, while miR-138-5p, miR-3656, and miR-638 did not show any significantly changes in their expression (Fig. 3f).

Based on these observations, 11 miRNAs were classified as synaptosomal miRNAs and two miRNAs as cytosolic miRNAs in the AD state.

### MicroRNAs expression in AD cytosol vs UC cytosol

Next, we compared AD cytosolic vs UC cytosolic miRNAs. A total of 13 hsa-miRNAs were found to be significantly deregulated in the AD cytosol vs UC cytosol comparison Supplementary Table 6. Interestingly, expression levels of all miRNAs were reduced in AD cytosol as mentioned in Supplementary Table 6. Supplementary Fig. 4a shows the hierarchical clustering and heatmap of significantly deregulated miRNAs with their ID numbers. The 13 miRNAs were found to be downregulated significantly (Supplementary Fig. 4b). Gene-filter analysis of total miRNAs pool showed that 99.76% of miRNA population did not show a significant difference in the cytosol vs synaptosome compartment. Only, 0.24% of miRNA populations showed variable expression levels. All 0.24% miRNA population is downregulated (Supplementary Fig. 4c). The scattered plot showed the average log2 fold changes values of significantly deregulated miRNAs (Supplementary Fig. 5a) and volcano plot showed the p values (−log10) of significantly deregulated miRNAs in AD cytosol vs AD cytosol (Supplementary Fig. 5b). The top candidate miRNAs were selected for validation analysis.

### Validation analysis of cytosolic miRNAs in AD and unaffected control

*(i) AD and UC postmortem brains:* Validation analysis of cytosolic miRNAs were performed on 15 UC and 27 AD postmortem brain samples. The 13 miRNAs candidates were selected for validation analysis. Opposed to the Affymetrix data, nine miRNAs were significantly upregulated in AD cytosol relative to UC cytosol and three miRNAs did not show significant changes (Supplementary Fig. 4d). Again, the differences in the validation data could be due to sample-to-sample pathological and genetic variations.

*(ii) WT, APP-Tg, and Tau-Tg mice:* We also performed the validation of cytosolic miRNAs in APP-Tg and Tau-Tg mice relative to WT mice. Other than the 13 cytosolic mmu-miRNAs, we also checked the expression of other potential mmu-miRNAs: miR-17-5p, let-7d-5p, miR-185-5p, miR-103-3p, miR-138-5p, miR-877-5p, miR-24-3p, miR-502-3p, miR-140-3p, miR-124-3p, and miR-3656. Most of the miRNAs were upregulated in the APP-Tg and Tau-Tg cytosol relative to WT cytosol. Only, miR-638 and miR-3656 were significantly downregulated in APP-Tg cytosol relative to WT (Supplementary Fig. 6).

### MicroRNAs expression in AD synaptosomes vs UC synaptosomes

Lastly, we compared the microarray data for miRNAs expression changes in AD synaptosomes vs UC synaptosomes. A total of 11 miRNAs were found to be deregulated significantly in AD synaptosomes vs UC synaptosomes comparison as shown in (Supplementary Table 7). Four hsa-miRNAs- miR-502-3p, miR-500a-3p, miR-877-5p, and miR-664b-3p were highly expressed in AD synaptosomes relative to UC synaptosomes. The remaining seven hsa-miRNAs—miR-3196, miR-6511b-5p, miR-4508, miR-1237-5p, miR-5001-5p, miR-4492, and miR-4497 showed reduced expression in AD synaptosomes and were highly expressed in UC synaptosomes. The differential expression of these miRNAs in AD and UC synaptosomes suggests their importance in synapse function.

**Fig. 4:**
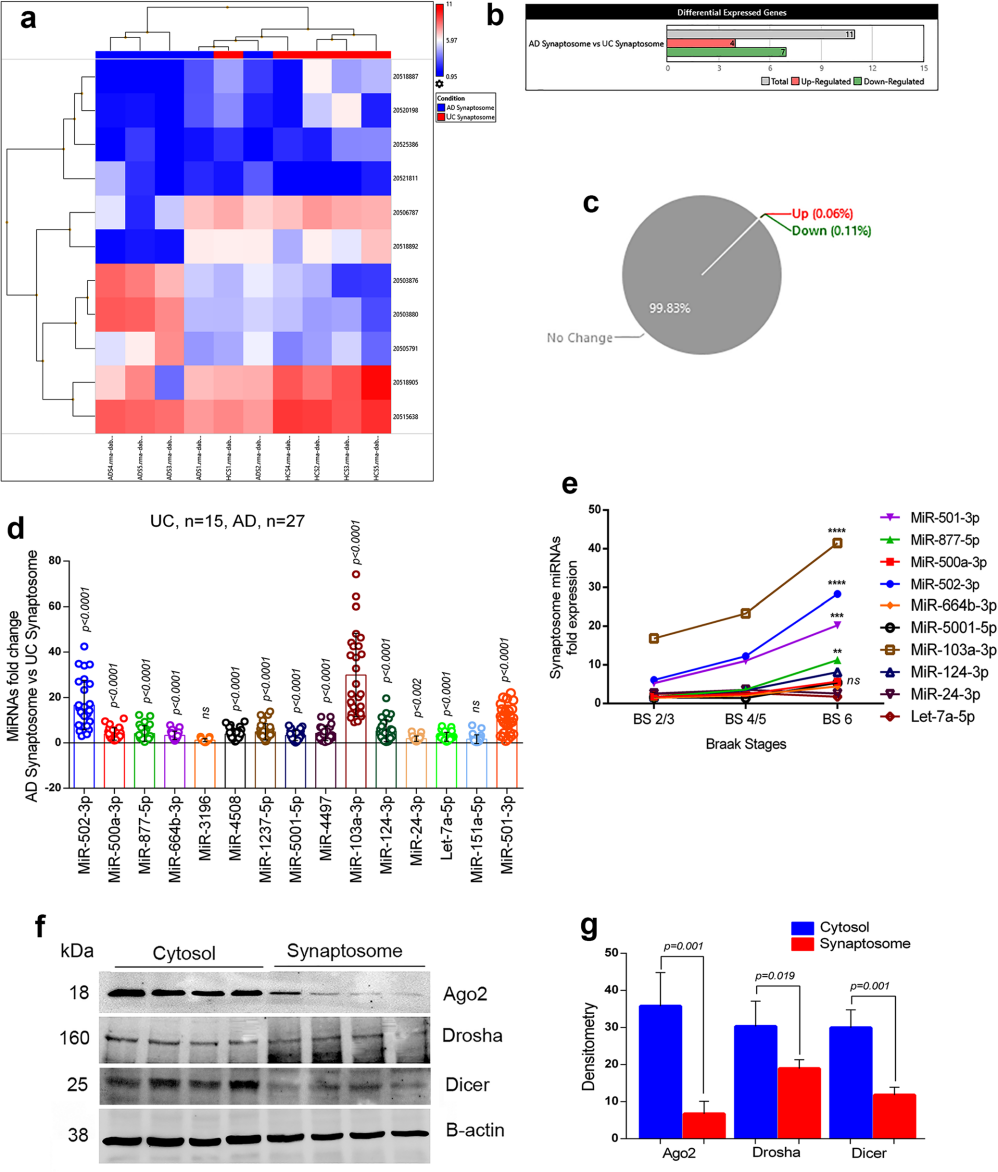
MiRNAs expression in synaptosome in AD and healthy state. **a** Hierarchical clustering and heatmap of significantly deregulated miRNAs in synaptosome in AD and unaffected controls. (red color intensity showed the miRNAs upregulation and blue color intensity showed the miRNAs downregulation) **b** Total numbers of miRNAs deregulated in AD synaptosome vs UC synaptosome. (grayscale bar—total number of miRNAs; red scale bar—upregulated miRNAs; green scale bar—downregulated miRNAs). **c** Pi diagram showed the total miRNAs pool distribution and percentage of miRNAs population changed in AD synaptosome vs UC synaptosome. **d** qRT-PCR-based validation analysis of significantly deregulated miRNAs in AD (*n* = 27) and UC (*n* = 15) synaptosome. MiRNAs expression was quantified in terms of fold changes in AD synaptosome relative to UC synaptosome. Each circle dot represents one sample. **e** Multiple comparison analysis of synaptosomal miRNAs fold changes with Braak stages 2/3, Braak stages 4/5 and Braak stages 6 of AD samples. (**P < 0.01, ***P < 0.001, ****P < 0.0001). **f** Immunoblotting analysis of miRNAs biogenesis proteins (Ago2, Drosha, and Dicer) in the cytosol and synaptosomal of UC samples (*n* = 4). **g** Densitometry analysis of Ago2, Drosha and Dicer in cytosol relative to synaptosomes of UC samples. All blots are driven from the same experiment and were proceeded parallelly (**f**). Values in the bar diagrams are mean ± SEM and error bars are equivalent throughout the figure (**d**, **g**).

Figure 4a showed the hierarchical clustering and heatmap of significantly deregulated miRNAs with their ID numbers. The four miRNAs were upregulated, and seven miRNAs were downregulated significantly (Fig. 4b). Gene-filter analysis of total miRNAs pool showed that 99.83% of the miRNA population did not show a significant difference in the synaptosome compartments in AD vs UC. Only 0.17% miRNAs populations showed variable expression patterns. The 0.06% of miRNAs is upregulated and 0.11% of the miRNA population is downregulated (Fig. 4c). The scattered plot showed the average log2 fold changes values of significantly deregulated miRNAs (Supplementary Fig. 5c) and the volcano plot showed the *P* values (−log10) of significantly deregulated miRNAs in AD synaptosomes vs UC synaptosomes (Supplementary Fig. 5d). The top candidate miRNAs were selected for validation analysis.

Based on the miRNAs’ expression pattern in cytosol and synaptosomes in AD vs UC samples, 15 miRNAs (68.2%) were expressed only in the cytosol, and seven miRNAs (31.8%) were expressed only in the synaptosomes. We did not see any miRNA that were commonly expressed in both conditions.

### Validation analysis of synaptosomal miRNAs

*(i) AD and UC postmortem brains****:*** Validation analysis were performed on 15 UC and 27 AD postmortem brains. We checked synaptosomal expression of deregulated 16 miRNAs. However, only 14 hsa-miRNAs were amplified, the 12 hsa-miRNAs (miR-502-3p, miR-500a-3p, miR-877-5p, miR-664b-3p, miR-4508, miR-1237-5p, miR-5001-5p, miR-4497, miR-103a-3p, miR-124-3p, miR-24-3p, and let-7a-5p were significantly upregulated in the AD synaptosomes relative to UC synaptosomes, while two hsa-miRNAs (miR-3196 and miR-151-5p) did not show any significant changes (Fig. 4d).

*(ii) WT, APP-Tg, and Tau-Tg mice****:*** We also performed the validation of the above-mentioned miRNAs and other potential synaptosomal miRNAs in APP-Tg and Tau-Tg mice relative to WT mice. The 12 mmu-miRNAs, which were, amplified successfully included- miR-17-5p, let-7d-5p, miR-185-5p, miR-103-3p, miR-138-5p, miR-877-5p, miR-24-3p, miR-502-3p, miR-140-3p, miR-124-3p, miR-638, and miR-3656. In APP-Tg mice synaptosomes, seven miRNAs were significantly upregulated, four were significantly downregulated relative to WT synaptosomes and one miRNA showed no change (Supplementary Fig. 6). In Tau-Tg synaptosomes, nine miRNAs were significantly upregulated, and three miRNAs were significantly downregulated relative to WT synaptosomes (Supplementary Fig. 6).

Summarizing all validation analysis, only 12 miRNAs expression was consistent in different comparisons and sample settings. The ten miRNAs can be classified as synaptosomal miRNAs and two miRNAs as cytosolic miRNAs. The other miRNAs expression patterns were not aligned with Affymetrix data and qRT-PCR validation. This could be due to variation of the Braak stages of postmortem AD brains used for Affymetrix analysis and qRT-PCT validation.

Next, we examined the synaptosomal miRNAs expression patterns with AD samples Braak stages. Multiple comparison analyses showed that the expression of synaptosomal miRNAs were gradually increased with Braak stages. However, significant differences were found in miR-501-3p (*P* = 0.001), miR-502-3p (*P* < 0.0001), miR-877-5p (*P* = 0.010), and miR-103a-3p (*P* < 0.0001) fold changes at Braak stage 6 relative to Braak stage 2/3 (Fig. 4e). These results unveiled the strong connection of these miRNAs with AD progression.

Further, to determine the synaptosomal miRNAs synthesis at the synapse, we checked the levels of key miRNA biogenesis proteins (Ago2, Drosha, and Dicer) in the cytosol and synaptosome fractions. In Fig. 4f, immunoblots showed the levels of miRNA biogenesis proteins in UC cytosol and synaptosomes. Densitometry analysis showed very high levels of all three proteins in cytosol relative to synaptosomes (Fig. 4g). The presence of miRNA biogenesis proteins in synaptosomes confirmed that miRNAs might be synthesized at the synapse.

### In silico ingenuity® pathway analysis of cytosolic and synaptosomal miRNAs in AD and healthy state

The deregulated miRNAs under different conditions were run for IPA analysis. The first comparison was cytosolic vs synaptosomal miRNAs in the healthy state. The top deregulated miRNAs were involved in several diseases, molecular and cellular functions, physiological system development and functions (Supplementary Data 1). However, we focused on the miRNA candidates which are involved in nervous system development and function in neurological diseases. Eleven miRNAs were identified which were significantly (*P* < 0.05) involved in many neurological diseases and dementia, including AD and MCI (Supplementary Fig. 7a). Next, we analyzed the mRNA target and seed sequences of these miRNAs to understand the molecular mechanism of miRNAs involved in neurological function (Supplementary Fig. 7b). The tumor suppressor gene (TP53) was the central gene that was targeted by many of these miRNAs. Other potential genes were BACE1, Smad2/3, Lypla1, Akt1, and SERBP1 pathway genes.

**Fig. 5:**
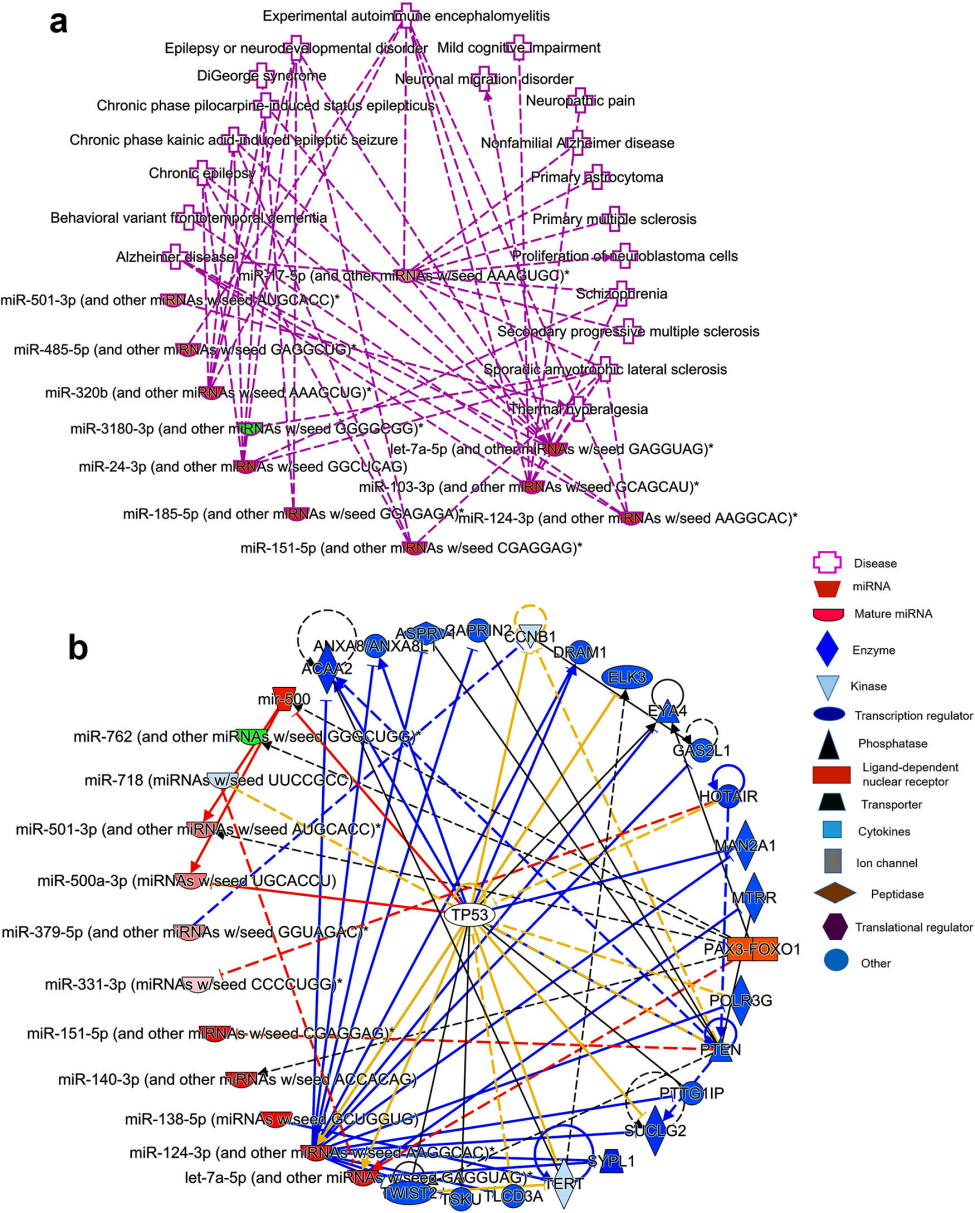
Ingenuity pathway analysis of cytosolic and synaptosomal miRNAs in AD. **a** In AD state, cytosolic and synaptosomal miRNAs expression network in various human diseases. Red nodes represent increased expression and green nodes represent a decreased expression of miRNAs. **b** MiRNAs target and seed sequences network of cytosolic and synaptosomal miRNAs in the AD state.

Similarly, we studied synaptosomal and cytosolic miRNAs function in AD cases. The top miRNA candidates were significantly (*P* < 0.05) involved in several nervous system development, function, and neurological diseases (Supplementary Data 2). However, our interest was neurological disorders and dementia, where eight miRNAs were detected which were involved in several neurological disorders, including AD (Fig. 5a). Further, miRNAs target predication analysis showed more than 20 genes that are targeted by these miRNAs (Fig. 5b). Next, we studied the biological roles of cytosolic miRNAs which were downregulated in AD compared to UC. The top five miRNAs were significantly involved in several diseases and molecular pathways (Supplementary Data 3). MiRNAs and diseased pathways showed integration with Amyotrophic lateral sclerosis (Supplementary Fig. 8a). Like other miRNAs, several genes were identified as a potential target for these five miRNAs (Supplementary Fig. 8b).

**Fig. 6:**
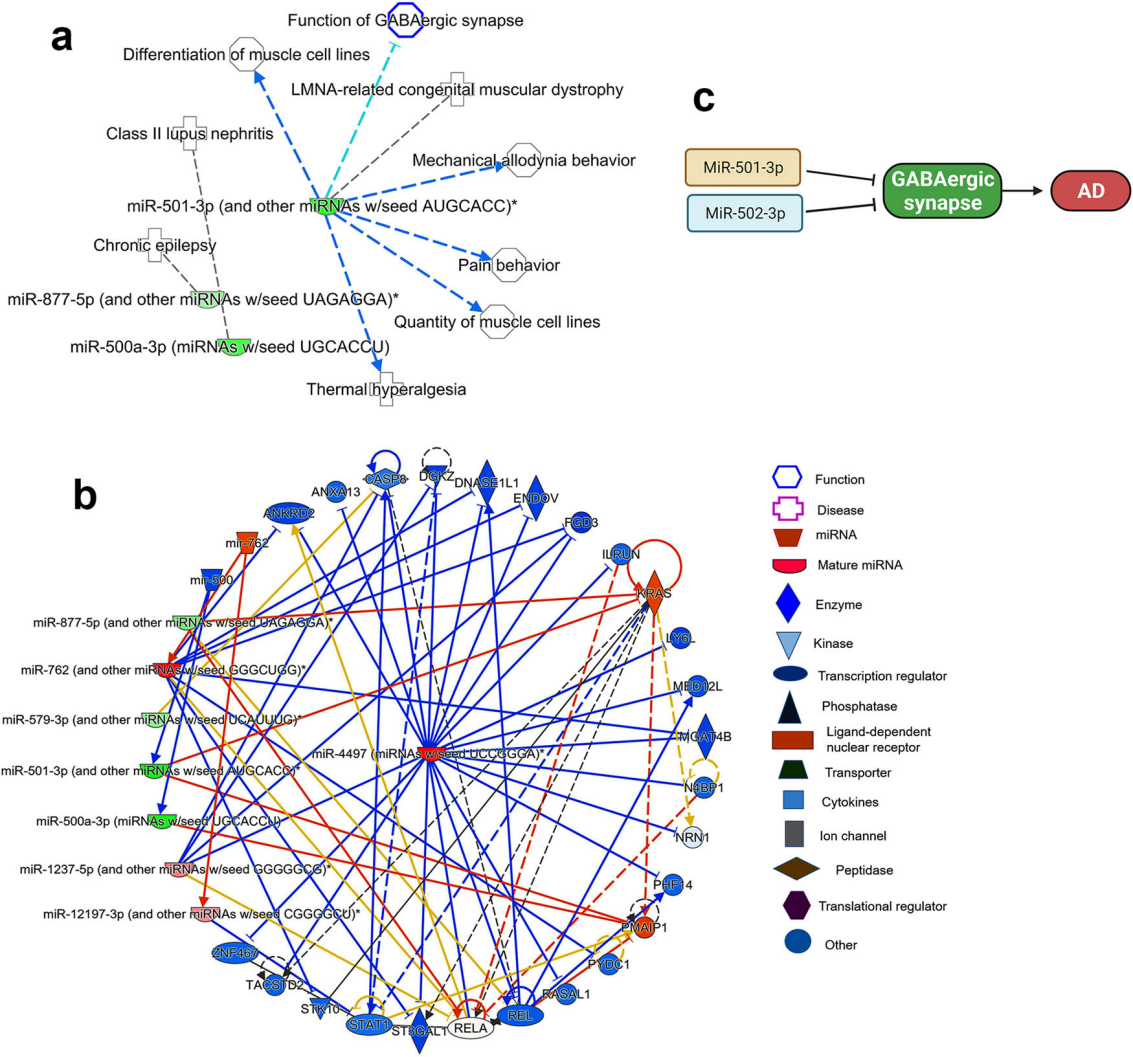
Ingenuity pathway analysis of synaptosomal miRNAs in AD. **a** Synaptosomal miRNAs expression network in various human diseases. **b** MiRNAs target and seed sequences network of synaptosomal miRNAs in the AD and healthy state. Red nodes represent increased expression and green nodes represent a decreased expression of miRNAs. **c** Possible molecular mechanism of miR-501-3 and miR-502-3p in AD progression via negative modulation of GABAergic synapse. Inhibition of GABARA1 expression by the overexpression of these miRNAs could inhibits the GABAergic synapse function in AD.

Lastly, we studied the biological functions of synaptosomal miRNAs which were deregulated in AD vs UC. The miR-500 family (miR-501-3p, miR-500a-3p) and miR-877-5p were identified to be significantly (*P* < 0.05) involved in several biological processes and disorders (Supplementary Data 4). MiRNA and disease interaction analysis showed a significant connection of miR-501-3p in GABAergic synapse function and other brain functions (Fig. 6a). The miRNAs target predication analysis showed more than 20 genes that are targeted by these miRNAs (Fig. 6b). The GABARA1 gene was identified as one of the potential common target of miR-501-3p and miR-502-3p (Supplementary Fig. 9). Further, gene ontology enrichment analysis of miR-502-3p showed that it involved in several biological processes, cellular components, and molecular functions. The most significant involvement was a response to external stimuli (*P* = 0.009) and nervous system development (*P* = 0.044). The most significant cellular component was GABAergic synapse (*P* = 0.028), and the molecular function was calmodulin binding (*P* = 0.020) (Supplementary Fig. 10).

Overall, IPA and gene ontology enrichment analyses showed that synaptosomal miRNAs are altered in several neurological disorders and participate in numerous cellular and molecular pathways related to brain function.

## Discussion

Synaptosome-based research in AD began since the discovery of the synaptosome by Hebb and Whittaker in 1958^51^. Although significant research has been done on synaptosomal function/dysfunction, we still know very little about physiological connections and pathological changes in AD, particularly the sequence of events that occur at the synapse and the regulation of miRNAs in synaptosomes and how synaptosomal miRNAs are different from cytosolic miRNAs.

Using global synaptosomal and cytosolic miRNA analysis, in silico analysis, transmission electron microscopy of healthy unaffected and AD postmortem brains and brain tissues from APP and Tau transgenic mice, in the current study we investigated a comprehensive synaptic and cytosolic miRNAs analysis. We also determined the possible molecular function of synaptic miRNAs in AD and brain aging.

It is well-studied that miRNAs are present in different cell organelles and cellular components such as the nucleus, mitochondria, Golgi bodies, exosomes and apoptotic bodies. These differentially expressed miRNAs, can modulate the levels of localized proteins^52^. Therefore, we hypothesized that synapse-centered miRNAs are altered in AD. We also hypothesize that miRNAs in synaptosomes and cytosols are “differently expressed and localized” in healthy (unaffected controls) and AD states. Therefore, for the first time, our study distinguished cytosolic and synaptosomal miRNAs and their alterations in healthy and AD states.

We examined cytosolic and synaptosomal miRNAs changes in both healthy and disease states. In primary screenings, some individual synaptosomal and cytosolic miRNAs were identified as those which were expressed in both healthy and disease states but with varying expression levels, in terms of fold change (≤ −2 and ≥ 2). We noted that fold change of similar synaptosomal miRNAs varied by >100-folds in AD relative to healthy state. Most of these synaptosomal miRNAs are studied in human diseases, but very limited information is available on the cytosolic miRNAs.

Interestingly, as shown by pie chart analysis, >99% of miRNAs population did not show significant changes in the synaptosome and cytosol. Only a small fraction (<1%) of miRNA pool showed significant changes among synaptosomes and cytosol populations. These findings confirmed that most of the miRNA populations are uniformly distributed in the neuron with an exception of some localized synapse miRNAs. These synaptosomal miRNAs are either synthesized locally at the synapse or may be transported from the soma to the synapse. As per our initial analysis, it seems that miRNA biogenesis machinery is present at the synapse, and it is possible that miRNAs processing occurs at the synapse. However, additional research is needed to confirm miRNA biogenesis at the synapse.

Validation analysis on the postmortem brains and brain tissues from AD mouse models amplified only limited numbers of miRNAs compared to primary Affymetrix screening. Our extensive and careful validation analysis of postmortem brains revealed several potential miRNAs that showed similar expression trends specified as synaptosomal or cytosolic miRNAs in both healthy and AD states. Further, extended validation analysis of APP-Tg and Tau-Tg mice shortlisted quite a few specific miRNAs. We noticed that Affymetrix-based miRNA analysis did not agree 100% with qRT-PCR validation. It could happen because we conducted Affymetrix analysis using Braak stage VI-postmortem AD brains, and in our qRT-PCR validation, we used postmortem AD brains with all Braak stages and heterogeneity nature of AD samples obtained from three different NIH NeuroBioBanks. Further, we found the variation in synaptosomal miRNAs expression pattern in postmortem AD brain vs AD mice brain. It is well known that AD pathobiology is much more complex in human brain than AD mice. In mice, AD pathology is usually caused by only one mutation (APP or Tau) while in human there are multiple factors that contribute to AD progression. Hence, changes in the synaptosomal miRNAs levels depends on many aspects in human that may not be the case in AD mice. Irrespective of all these factors, some potential miRNAs showed consistent expression and agreeing with Affymetrix and qRT-PCR validation analysis in human and mice. Overall, human and mouse data analyses revealed ten potential miRNAs designated as synaptosomal miRNAs shown in Supplementary Fig. 6 are actively involved in several neural functions^53–58^.

Interesting data was obtained in the case of cytosolic miRNAs in AD vs healthy controls. The initial screening showed reduced expression of all cytosolic miRNAs in AD cytosol. This could be because of higher Aβ and p-tau concentrations in the cytoplasm compared to the synapse and high toxicities may be responsible for altered expression of miRNAs. Our careful validation analysis using postmortem brains, WT mice, APP-Tg and Tau-Tg mice strongly unveiled miR-638 and miR-3656 as potential cytosolic miRNAs. Both miRNAs are unique in AD and need further investigation on cytosolic basis of AD progression.

The top synaptosomal miRNAs are miR-500a-3p, miR-501-3p, miR-502-3p, and miR-877-5p. In addition, the most downregulated miRNA was miR-4499 as shown by the primary screening. MiR-500 cluster miRNAs were amplified in all validation settings; however, we did not see any significant expression of miR-4499 in the validation phase. The Gene Ontology Enrichment and IP analysis for the miR-500 cluster showed that miR-500 family is involved in key biological process, cellular function and molecular function.

The most significant biological process is response to external stimulus and the most significant cellular component is GABAergic synapse (Supplementary Fig. 10). GABAergic synapse is a crucial inhibitory synapse that is dysfunctional in AD (28,29,30,31). Our results also confirmed reduced levels of GABRA1 in AD synaptosomes. Further, in silico analysis showed that miR-502-3p could modulate the function of GABAergic synapse. Both Gene Ontology and IP analysis confirmed the strong links of miR-501-3p and miR-502-3p in GABAergic synapse pathways. It could be mediated via modulation of the GABAergic receptor genes by these miRNAs (Fig. 6c). Further, miR-501-3p and miR-502-3p expression was significantly increased with Braak stages of AD postmortem brains again confirming the strong connection of these miRNAs with AD. Therefore, more research is warranted to study the roles of miR-501-3p and miR-502-3p in the regulation of excitatory and inhibitory synapse function in relation to AD.

In summary, our study identified the synaptosomal miRNAs that are deregulated in AD. Our comprehensive analysis identified the three most promising synaptosomal miRNAs- miR-501-3p and miR-502-3p that could modulate the function of excitatory and inhibitory synapses in AD. Our ongoing research investigating the underlying molecular mechanism of miR-501-3p and miR-502-3p in synaptic activity and GABAergic synapse function in relation to Aβ and p-tau induced toxicities.

## Methods

### Postmortem brain samples

Postmortem brains from AD patients and unaffected controls were obtained from NIH NeuroBioBanks: (1) Human Brain and Spinal Fluid Resource Center, 11301 Wilshire Blvd (127A), Los Angeles, CA. (2) Brain Endowment Bank, University of Miami, Millar School of Medicine, 1951, NW 7th Avenue Suite 240, Miami, FL. (3) Mount Sinai NIH Brain and Tissue Repository, 130 West Kingsbridge Road Bronx, NY^59^. Brain tissues were dissected from the Brodmann’s Area 10 of the frontal cortices from AD patients (*n* = 27) and age- and sex-matched unaffected controls (*n* = 15). Demographic and clinical details of study specimens are provided in Supplementary Table 1. The study was conducted at the Internal Medicine Department, Texas Tech University Health Sciences Center, and Institutional Biosafety Committee (IBC protocol #14013) approved the study protocol for the use of human postmortem brain tissues obtained from NIH NeuroBioBank. The NIH NeuroBioBanks mentioned above are operated under their institution’s IRB approval, and they obtained written informed consent from the donors.

### Synaptosomes extraction

Synaptosomes were extracted using Syn-PER Reagent as per manufacturer instructions with some modifications (Thermo Scientific, USA)^48,49,60^. Briefly, 50 mg of brain tissue was used from each sample for synaptosome extraction in 1 ml of Syn-PER Reagent. Tissues were homogenized slowly by Dounce glass homogenization on ice with ~10 slow strokes. The resulting tissue homogenates were transferred to a centrifuge tube. Samples were centrifuged at 1400×*g* for 10 minutes at 4 °C to remove the leftover tissue debris. After centrifugation, the supernatant was transferred to a new tube. Again, supernatant (homogenate) was centrifuged at high-speed 15,000 × *g* for 20 min at 4 °C. The supernatant was removed as a cytosolic fraction and synaptosomes recovered in the pellet form. Both the cytosolic fraction and synaptosome pellet were processed for RNA and protein extraction. The synaptosome pellet was also processed for transmission electron microscopic (TEM) analysis.

### Synaptosomes characterization

Synaptosome preparations (purity and integrity) were characterized by TEM analysis of synapse assembly, immunoblotting of synaptic proteins—synapse associate protein 25 (SNAP25), postsynaptic density protein 95 (PSD95), and synaptophysin, and qRT-PCR analysis of similar synaptic genes^61,62^.

### Transmission electron microscopy of synaptosomes

Freshly isolated synaptosomes were processed for TEM analysis. Briefly, the pellet was fixed in a solution of 0.1 M cacodylate buffer, 1.5% paraformaldehyde, and 2.5% glutaraldehyde and then post-fixed with 1% osmium tetroxide and embedded in LX-112 resin. Ultrathin sections were cut, stained with uranyl acetate and lead citrate, and examined with the Hitachi H-7650 /Transmission Electron Microscope at 60 kV located at the College of Arts and Sciences Microscopy, Texas Tech University. Low-magnification imaging was followed by high-magnification imaging. Representative images were acquired and recorded with an AMT digital camera^63^.

### Immunoblotting analysis

We performed immunoblot analysis for the synaptic/cytosolic proteins, brain cells, and miRNAs biogenesis proteins. Details of the proteins and antibody dilutions are given in Supplementary Table 2. The 40 µg of protein lysates were resolved on a 4–12% Nu-PAGE gel (Invitrogen). The resolved proteins were transferred to nylon membranes (Novax Inc., San Diego, CA, USA) and then incubated for 1 h at room temperature with a blocking buffer (5% dry milk dissolved in a TBST buffer). The nylon membranes were incubated overnight with the primary antibodies SNAP25 (Novus Biologicals; NB100-1492), PSD95 (Novus Biologicals; NB300-556), Synaptophysin (Novus Biologicals; NB300-653), PCNA (Santa Cruz; sc-25280), elF2a (Cell Signaling; 2103), GABAR1a (Bioss Antibodies; bs-1232R), VGLUT1 (Thermofisher; 48-2400), NeuN (Abcam; ab177487), Iba1 (Cell Signaling; 17198), AGO2 (Cell Signaling; 2897), Drosha (Cell Signaling; 3364), Dicer (Thermofisher; PA5-115124), APP 6E10 (Biolegend; 803015), Phospho-Tau (Thermofisher; MN1020) and Beta-actin (Sigma; A2228). The membranes were washed with a TBST buffer three times at 10-min intervals and then incubated for 2 h with an appropriate secondary antibody, sheep anti-mouse HRP 1:10,000, followed by three additional washes at 10-min intervals. Proteins were detected with chemiluminescence reagents (Pierce Biotechnology, Rockford, IL, USA), and the bands from the immunoblots were visualized^63,64^. Source data are provided in the source data file.

### Quantitative real-time PCR analysis

Quantification of mRNA levels of synaptic genes was carried out with real-time qRT-PCR using methods described in ref. ^63^. The oligonucleotide primers were designed with primer express software (Applied Biosystems) for Synaptosomal- SNAP25, synaptophysin, PSD95, Eukaryotic translation initiation factor 1a (elF1a), and Proliferating cell nuclear antigen (PCNA). The primer sequences and amplicon sizes are listed in Supplementary Table 3. SYBR-Green chemistry-based quantitative real-time qRT-PCR was used to measure mRNA expression of these genes using β-actin as housekeeping genes, as described previously^63,65^.

### Affymetrix miRNA microarray analysis

Initially, we used five AD postmortem and five unaffected control (UC) postmortem brains for Affymetrix microarray analysis. The demographic and clinical details of samples used for Affymetrix analysis are given in Table 1. Total RNA was extracted from the synaptosomal and cytosolic fractions from both AD and unaffected control samples using the TriZol reagent with some modifications. Total we had 20 samples for miRNA analysis- AD synaptosome (*n* = 5), UC synaptosome (*n* = 5), AD cytosol (*n* = 5) and UC cytosol (*n* = 5). Detailed miRNAs screening of the synaptosome and cytosolic miRNAs were conducted at the University of Texas Southwestern Medical Center, Genomics and Microarray Core Facility, Dallas. The miRNA expression profiles were generated with Affymetrix GeneChip miRNA array v. 4.0.

**Table 1:** Demographic and clinical details of postmortem brains used for Affymetrix microarray analysis.

S. no.	Barcode	Age	Sex	Disease status	Race	Brain region	CDR	Braak score	PMI (hours)	Cause of death
1	77423	79	F	AD	W	BM-10	3	6	6.50	Coronary artery disease
2	77424	69	M	AD	W	BM-10	3	6	5.42	SEPSIS
3	77425	75	M	AD	W	BM-10	2	6	8.00	Respiratory failure
4	77426	94	F	AD	W	BM-10	5	6	4.33	Acute myocardial infraction
5	77427	82	M	AD	W	BM-10	5	6	20.67	Cardiorespiratory arrest
6	77428	65	M	Unaffected control	H	BM-10	0	0	3.83	Renal failure
7	77431	103	F	Unaffected control	W	BM-10	0	1	3.83	Lymphadenopathy
8	77433	75	M	Unaffected control	B	BM-10	0	1	5.00	Myocardial infarction
9	77436	93	M	Unaffected control	W	BM-10	0.5	0	4.17	Acute myocardial infraction
10	77437	84	F	Unaffected control	W	BM-10	0	1	5.48	Arteriosclerotic heart disease

### Microarray data analysis

Data were analyzed using four comparisons: (1) AD synaptosome vs AD cytosol, (2) unaffected control (UC) synaptosome vs UC cytosol, (3) AD cytosol vs UC cytosol, and (4) AD synaptosome vs UC synaptosome. Microarray data for miRNAs expression changes in synaptosomal vs cytosol fractions were analyzed using two main criteria’s- Gene-level fold change <−2 or >2 and Gene-level *P* value <0.05. A probe set (Gene/Exon) is considered expressed if ≥50% samples have detectable above background (DABG) values below DABG Threshold <0.05.

The GeneChip miRNA 4.0 arrays contain a 100% miRBase version 20 coverage: 30,424 mature miRNAs were from all organisms; 2578 from human, 1908 from mouse, and 728 from rat. The GeneChip miRNA 4.0 array demonstrated superior performance with 0.95 reproducibilities (inter- and intra-lot) and >80% of transcripts were detected at 1.3 amol from 130 ng of total RNA. Data were represented by the GeneChip miRNA 4.0 array in 4 logs that correlated with a dynamic range of >0.97 signal and >0.94-fold change.

Briefly, 8 ml of total RNA was treated for poly (A) tailing reaction at 37 °C for 15 min as per the protocol. A 4 ml of 5Flash Tag Biotin HSR ligation mix was added to poly (A) tailed RNA, and the mixture was incubated at 25 °C for 30 min, using the Flash Tag Biotin HSR Labeling kit following the manufacturer’s instructions (cat. no. HSR30FTA; Genisphere, LLC, Hatfield, PA, USA). Biotin HSR that labeled with RNA was mixed with an array hybridization cocktail according to the GeneChip. Eukaryotic Hybridization control kit manual and was processed using the Affymetrix GeneChip miRNA array. Samples were incubated on the hybridization array chip at 48 °C and 60 rpm for 16–18 h. After hybridization, the chips were washed and stained by GeneChip hybridization, washed again and then stained with an Affymetrix kit according to the manufacturer’s protocols. The hybridized chips were scanned with an Affymetrix GCS 3000 7G Scanner^66^.

Raw data were obtained, using the Affymetrix GeneChip array in the form of an individual CHP file. Each sample was then analyzed, using Transcriptome Analysis Console software v. 4. Tukey’s bi-weight average (log2) intensity was analyzed with an *P* value (<0.05) for both conditions, for all genes in the samples from AD and control group. SAM (significance analysis of microarray) with the R package was used to identify differentially expressed miRNA and gene probe sets in samples from the AD patients and the controls. Probe sets were considered biologically significant if the fold changes were less than minus two and more than plus two^66^. All miRNA microarray data are available at https://www.synapse.org/#!Synapse:syn26642975/files/.

### Validation of deregulated miRNAs using postmortem brains

The deregulated miRNAs obtained from Affymetrix analysis were further tested and validated on large number of AD postmortem brains (*n* = 27) and unaffected controls (*n* = 15). Validation of miRNAs was performed for four comparisons: (1) AD synaptosome vs AD cytosol, (2) UC synaptosome vs UC cytosol, (3) AD cytosol vs UC cytosol, and (4) AD synaptosome vs UC synaptosome. MiRNAs levels were quantified by using miRNAs qRT-PCR, which involved three steps (i) miRNAs polyadenylation, (ii) cDNA synthesis, and (iii) qRT-PCR as described previously^66–68^. Primers for desired miRNAs were synthesized commercially (Integrated DNA Technologies Inc., IA, USA) (Supplementary Table 3). To normalize the miRNA expression, U6 snRNA and sno-202 were used as internal controls. The reaction mixture of each sample was prepared in triplicates. The reaction was set in the 7900HT Fast Real-Time PCR System (Applied Biosystems, USA). qRT-PCR was performed in triplicate, and the data were expressed as the mean ± SD.

### Validation of differentially expressed miRNAs using AD mouse models

The deregulated miRNAs obtained from Affymetrix analysis were further validated using brain tissues from 12-month-old APP Transgenic (Tg2576) (*n* = 6), Tau transgenic (P301L) (*n* = 7) and age- and sex-matched wild-type (WT) (*n* = 7) mice. The deregulated miRNAs were conserved in both humans and mice. The APP-Tg, Tau-Tg, and WT mice were obtained from Jackson Laboratories and the colonies were maintained in our lab. This study was carried out in strict accordance with the recommendations of U.S. National Institutes of Health Guide for the Care and Use of Laboratory Animals. The Institutional Animal Care and Use Committee (IACUC approval #16007) approved the protocol. Mice were euthanized to extract brain tissues. The brains were dissected, and the cerebral cortex was used for cytosol and synaptosome miRNA extraction. Validation of miRNAs were performed for four comparisons: (1) AD mice synaptosome vs cytosol, (2) WT mice synaptosome vs cytosol, (3) AD mice cytosol vs WT mice cytosol, and (4) AD mice synaptosome vs WT mice synaptosome. MiRNAs levels in APP and Tau mice relative to WT mice were quantified by using miRNAs qRT-PCR.

### In silico analysis for potential miRNAs

The QIAGEN’s Ingenuity^®^ Pathway Analysis (IPA^®^, QIAGEN Inc., https://www.qiagenbioinformatics.com/products/ingenuity-pathway-analysis) program was used to analyze the synaptosomal and cytosolic miRNAs target genes with false discovery rate (FDR) *P* values <0.05 and with *P* value <0.05. The IPA was used to gain insight into the overall biological changes caused by the expression, target gene prediction for synaptosomal and cytosolic miRNAs with AD and unaffected controls and gene Integrated Analysis. Each gene was related to various functions, pathways, and diseases as analyzed using the Ingenuity knowledge base platform. The miRNA target genes (predicted and validated) were identified using various online miRNA algorithms (diana-microt, microrna.org, mirdb, rna22-has, targetminer, and targetscan-vert)^66,68^.

### Statistical considerations

Statistical parameters were calculated using Prism software, v6 (La Jolla, CA, USA). Results are reported as mean ± SD. The results were analyzed by two-tailed Student’s *t* test to evaluate miRNAs expression in two groups of samples: (1) AD synaptosome vs AD cytosol, (2) UC synaptosome vs UC cytosol, (3) AD cytosol vs UC cytosol, and (4) AD synaptosome vs UC synaptosome. One-way comparative analysis of variance was used for analyzing WT, APP-Tg and Tau-Tg mice synaptosome vs cytosolic miRNAs data. Significant differences in three group of samples were calculated by Bonferroni’s multiple comparison tests. The correlation of miRNAs fold changes with Braak stages was analyzed by Tukey’s multiple comparisons test. *P* < 0.05 was considered statistically significant.

### Reporting summary

Further information on research design is available in the Nature Research Reporting Summary linked to this article.

## Supplementary information

Reporting Summary Checklist

Supplementary information

Supplementary Data 1

Supplementary Data 2

Supplementary Data 3

Supplementary Data 4

## Data Availability

The microarray datasets of miRNAs have been deposited in the ArrayExpress database at EMBL-EBI (https://www.ebi.ac.uk/arrayexpress/) under ArrayExpress accession number E-MTAB-11983. The miRNA Affymetrix microarray data for all samples are also available at (https://www.synapse.org/#!Synapse:syn26642975/files/). The data can be accessed by using the Synapse ID: syn26642975. The other data generated from this study are available from the corresponding author on reasonable request.

## Code availability

Raw data were obtained using the Affymetrix GeneChip array 4.0 platform in the form of an individual CHP file. Each sample was then analyzed using Transcriptome Analysis Console software v. 4.
