# Supplementary material for: Synaptosome microRNAs regulate synapse functions in Alzheimer’s disease
Source: NPJ Genom Med. 2022 Aug 8;7:47. doi: 10.1038/s41525-022-00319-8 (PMC9359989; doi:10.1038/s41525-022-00319-8)
Supplement: Supplementary file 1 — Reporting Summary Checklist [file 41525_2022_319_MOESM1_ESM.pdf]

nature portfolio

Corresponding author(s): Subodh Kumar

Last updated by author(s): Jul 8, 2022

Reporting Summary

Nature Portfolio wishes to improve the reproducibility of the work that we publish. This form provides structure for consistency and transparency in reporting. For further information on Nature Portfolio policies, see our Editorial Policies and the Editorial Policy Checklist.

Statistics

For all statistical analyses, confirm that the following items are present in the figure legend, table legend, main text, or Methods section.

n/a	Confirmed	
☐	☒	The exact sample size (*n*) for each experimental group/condition, given as a discrete number and unit of measurement
☐	☒	A statement on whether measurements were taken from distinct samples or whether the same sample was measured repeatedly
☐	☒	The statistical test(s) used AND whether they are one- or two-sided<br>*Only common tests should be described solely by name; describe more complex techniques in the Methods section.*
☒	☐	A description of all covariates tested
☐	☒	A description of any assumptions or corrections, such as tests of normality and adjustment for multiple comparisons
☐	☒	A full description of the statistical parameters including central tendency (e.g. means) or other basic estimates (e.g. regression coefficient) AND variation (e.g. standard deviation) or associated estimates of uncertainty (e.g. confidence intervals)
☐	☒	For null hypothesis testing, the test statistic (e.g. *F*, *t*, *r*) with confidence intervals, effect sizes, degrees of freedom and *P* value noted<br>*Give P values as exact values whenever suitable.*
☒	☐	For Bayesian analysis, information on the choice of priors and Markov chain Monte Carlo settings
☒	☐	For hierarchical and complex designs, identification of the appropriate level for tests and full reporting of outcomes
☐	☒	Estimates of effect sizes (e.g. Cohen's *d*, Pearson's *r*), indicating how they were calculated

Our web collection on statistics for biologists contains articles on many of the points above.

Software and code

Policy information about availability of computer code

Data collection	Hitachi H-7650 /Transmission Electron Microscope used to acquire images. Affymetrix GeneChip miRNA array v. 4.0 used for microarray data collection.
Data analysis	Transcriptome Analysis Console V4 used for microarray data analysis. QIAGEN's Ingenuity® Pathway Analysis (IPA®, QIAGEN Inc., https://www.qiagenbioinformatics.com/products/ingenuity-pathway-analysis) used for informatics analysis. Prism software, v6 used for statistical data analysis.

For manuscripts utilizing custom algorithms or software that are central to the research but not yet described in published literature, software must be made available to editors and reviewers. We strongly encourage code deposition in a community repository (e.g. GitHub). See the Nature Portfolio guidelines for submitting code & software for further information.

Data

Policy information about availability of data

All manuscripts must include a data availability statement. This statement should provide the following information, where applicable:

- Accession codes, unique identifiers, or web links for publicly available datasets
- A description of any restrictions on data availability
- For clinical datasets or third party data, please ensure that the statement adheres to our policy

All microRNAs data are available at https://www.synapse.org/#!Synapse:syn26642975/files/. The other data generated from this study are available from the corresponding author on reasonable request.

Field-specific reporting

Please select the one below that is the best fit for your research. If you are not sure, read the appropriate sections before making your selection.

☒ Life sciences ☐ Behavioural & social sciences ☐ Ecological, evolutionary & environmental sciences

For a reference copy of the document with all sections, see nature.com/documents/nr-reporting-summary-flat.pdf

Life sciences study design

All studies must disclose on these points even when the disclosure is negative.

Sample size	For experiments involving initial microarray screening of miRNAs involved n=5 samples in each groups, and qRT-PCR quantification of miRNAs involved n=15 control and n=27 AD cases was sufficient for sample replicates. We determined this to be sufficiently include control and diseased samples for proper statistical analysis.
Data exclusions	Data were not excluded from analysis.
Replication	All replication attempts were successful and observed expression patterns of potential miRNAs were consistent. For final quantification, all samples were quantified for a minimum of three replicates.
Randomization	Transmission electron microscopic images of synaptosomes from AD and healthy controls were selected randomly.
Blinding	Blinding was not possible as experimental conditions were evident from the image data. Quantifications were performed using computational pipeline applied equally to all conditions and replicates for a given experimental group.

Reporting for specific materials, systems and methods

We require information from authors about some types of materials, experimental systems and methods used in many studies. Here, indicate whether each material, system or method listed is relevant to your study. If you are not sure if a list item applies to your research, read the appropriate section before selecting a response.

Materials & experimental systems

n/a	Involved in the study	
☐	☒	Antibodies
☒	☐	Eukaryotic cell lines
☒	☐	Palaeontology and archaeology
☐	☒	Animals and other organisms
☒	☐	Human research participants
☒	☐	Clinical data
☒	☐	Dual use research of concern

Methods

n/a	Involved in the study	
☒	☐	ChIP-seq
☒	☐	Flow cytometry
☒	☐	MRI-based neuroimaging

Antibodies

Antibodies used

Summary of antibody dilutions and conditions used in the immunoblotting analysis of synaptosomes are provided in SI Table 2.
Marker Primary Antibody – Species and Dilution Purchased from Company, City & State Secondary Antibody, Dilution Purchased from Company, City & State.
PSD95 Rabbit Monoclonal 1:300 Novus Biological, Littleton, CO Donkey Anti-rabbit HRP 1:10,000, SNAP25 Rabbit, Polyclonal 1:500 Novus Biological, Littleton, CO Donkey. SYN Rabbit Polyclonal 1:500 Novus Biological, Littleton, CO. PCNA Mouse Monoclonal 1:500 Santa Cruz Biotechnology, Inc., Dallas, TX. elF2a Mouse Monoclonal 1:500 Cell Signaling Technology, Danvers, . GABAR1a Rabbit Polyclonal 1:500 Bioss Antibodies, Woburn, MA. VGLUT1 Rabbit Polyclonal 1:500 Thermo Fisher Scientific, Waltham. NueN Rabbit Monoclonal 1:1000 Abcam, Cambridge, UK. IBa1/AIF-1 Rabbit Monoclonal 1:1000 Cell Signaling Technology, Danvers, MA. AGO2 Rabbit Monoclonal 1:500 Cell Signaling Technology, Danvers, MA. Drosha Rabbit Monoclonal 1:500 Cell Signaling Technology, Danvers. Dicer Rabbit Polyclonal 1:500 Thermo Fisher Scientific, Waltham. 6E10 Mouse Monoclonal 1:500 Biolegend, San Diego, CA Sheep Anti-mouse HRP 1:10,000 GE Healthcare Amersham, Piscataway, NJ. p-Tau (HT7) Mouse Monoclonal 1:500 Thermo Fisher Scientific, Waltham, MA. B-actin Mouse Monoclonal 1:500 Sigma-Aldrich St Luis, MO Sheep anti-mouse HR 1:10,000 GE Healthcare Amersham Piscataway, NJ.

Antibodies sources and catalogue numbers- SNAP25 (Novus Biologicals; NB100-1492), PSD95 (Novus Biologicals; NB300-556), Synaptophysin (Novus Biologicals; NB300-653), PCNA (Santa Cruz; sc-25280), elF2a (Cell signaling; 2103), GABAR1a (Bioss Antibodies; bs-1232R), VGLUT1 (Thermofisher; 48-2400), NeuN (Abcam; ab177487), Iba1 (Cell signaling; 17198), AGO2 (Cell signaling; 2897), Drosha (Cell signaling; 3364), Dicer (Thermofisher; PA5-115124), APP 6E10 (Biolegend; 803015), Phospho-Tau (Thermofisher; MN1020) and Beta-actin (Sigma; A2228).

Validation	Most of the antibodies (6E10, PSD95, SNAP25, Synaptophysin, NeuN, GFAP and B-actin) were validated in our previous studies (Kumar et al, Redox Biol. 2021 Nov 9;48:102182). The other antibodies p-Tau, Ago2, Drosha, Dicer, PCNA, elF2a has been validated by manufacturers by demonstrating immunoblotting on human and mouse brain and cell lines extract.

Animals and other organisms

Policy information about studies involving animals; ARRIVE guidelines recommended for reporting animal research

Laboratory animals	Our study includes 12-month-old APP Transgenic (Tg2576) (n=6), Tau transgenic (P301L) (n=7) and age and sex matched wild type (C57BL6) (n=7) mice obtained from Jackson Laboratories and the colonies were maintained in our lab.
Wild animals	No wild animals were used in this study.
Field-collected samples	No field-collected samples were used in this study.
Ethics oversight	The mouse work was performed under the study protocol (IACUC approval #16007), as approved by the Institutional Animal Care and Use Committee.

Note that full information on the approval of the study protocol must also be provided in the manuscript.
