# Supplementary material for: Synaptosome microRNAs regulate synapse functions in Alzheimer’s disease
Source: NPJ Genom Med. 2022 Aug 8;7:47. doi: 10.1038/s41525-022-00319-8 (PMC9359989; doi:10.1038/s41525-022-00319-8)
Supplement: Supplementary file 2 — Supplementary information [file 41525_2022_319_MOESM2_ESM.pdf]

Supplementary Information

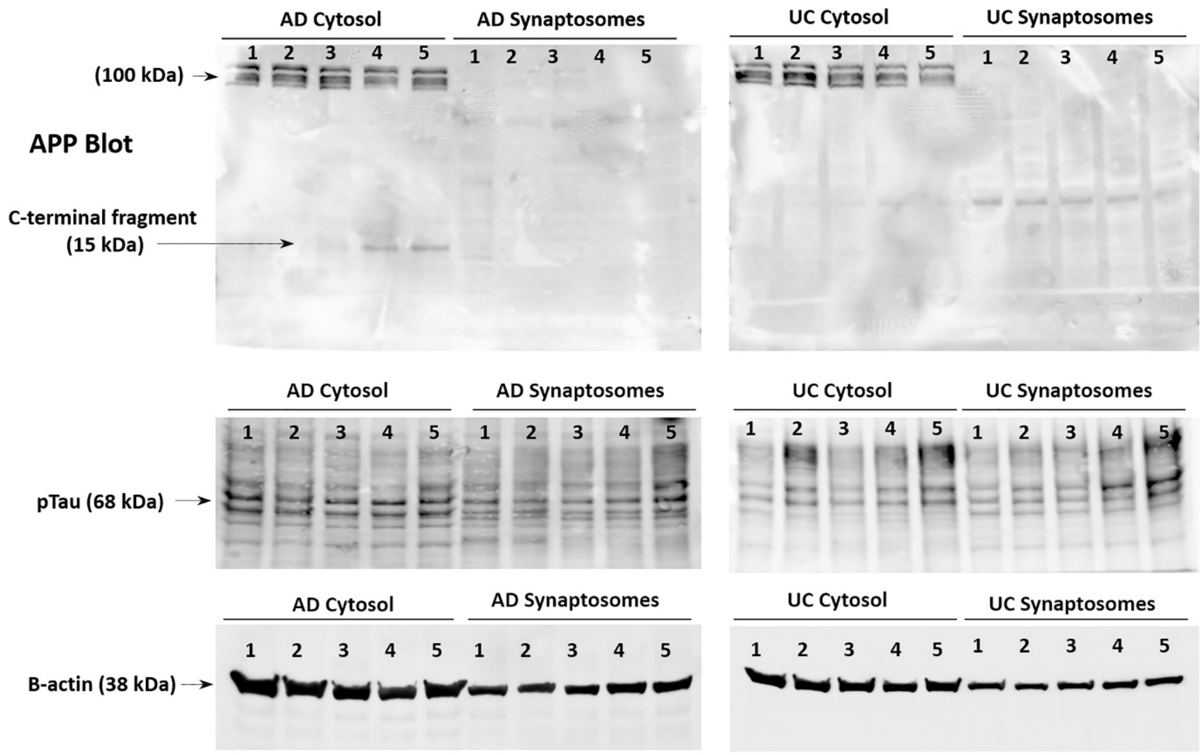

Supplementary Figure 1. Immunoblotting of APP and p-tau proteins in cytosol and synaptosome in AD and unaffected controls. All blots are driven from the same experiment and were proceed parallelly.

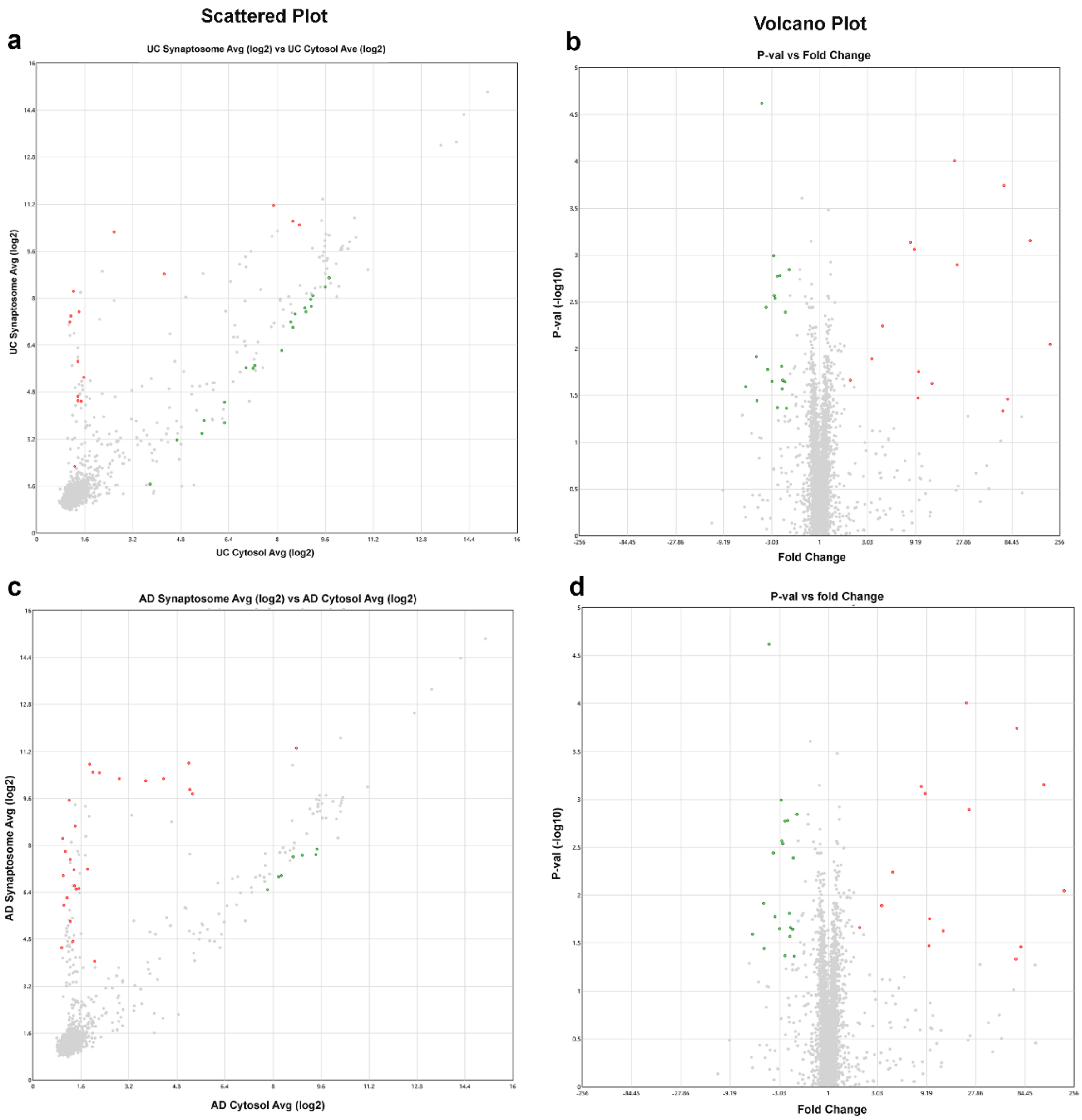

Supplementary Figure 2. (a) Scattered plot showing the average log2 fold changes values of significantly deregulated miRNAs in cytosol and synaptosome in healthy state. (b) Volcano plot showing the p values (-log10) of significantly deregulated miRNAs in cytosol and synaptosome in healthy state. (c) Scattered plot showing the average log2 fold changes values of significantly deregulated miRNAs in cytosol and synaptosome in AD state. (d) Volcano plot showing the p values (-log10) of significantly deregulated miRNAs in cytosol and synaptosome in AD state.

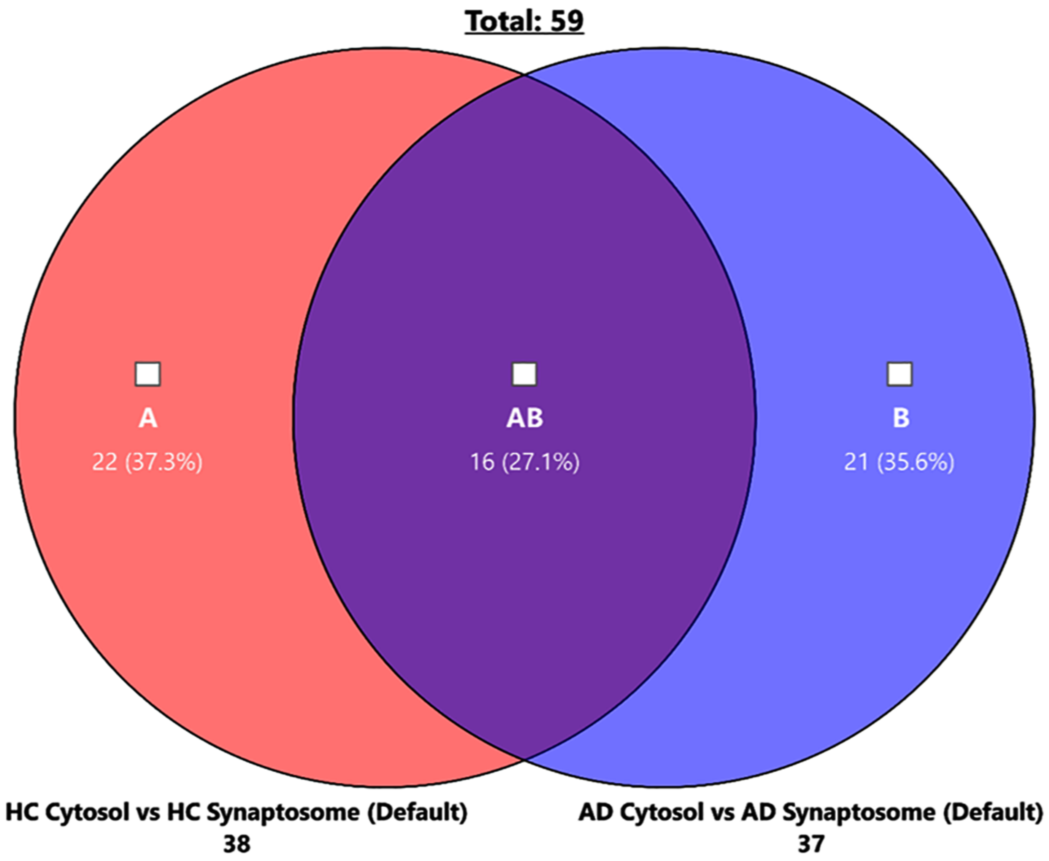

Supplementary Figure 3. Venn diagram showing the number of miRNAs that expressed only in cytosol and synaptosome in AD vs healthy state.

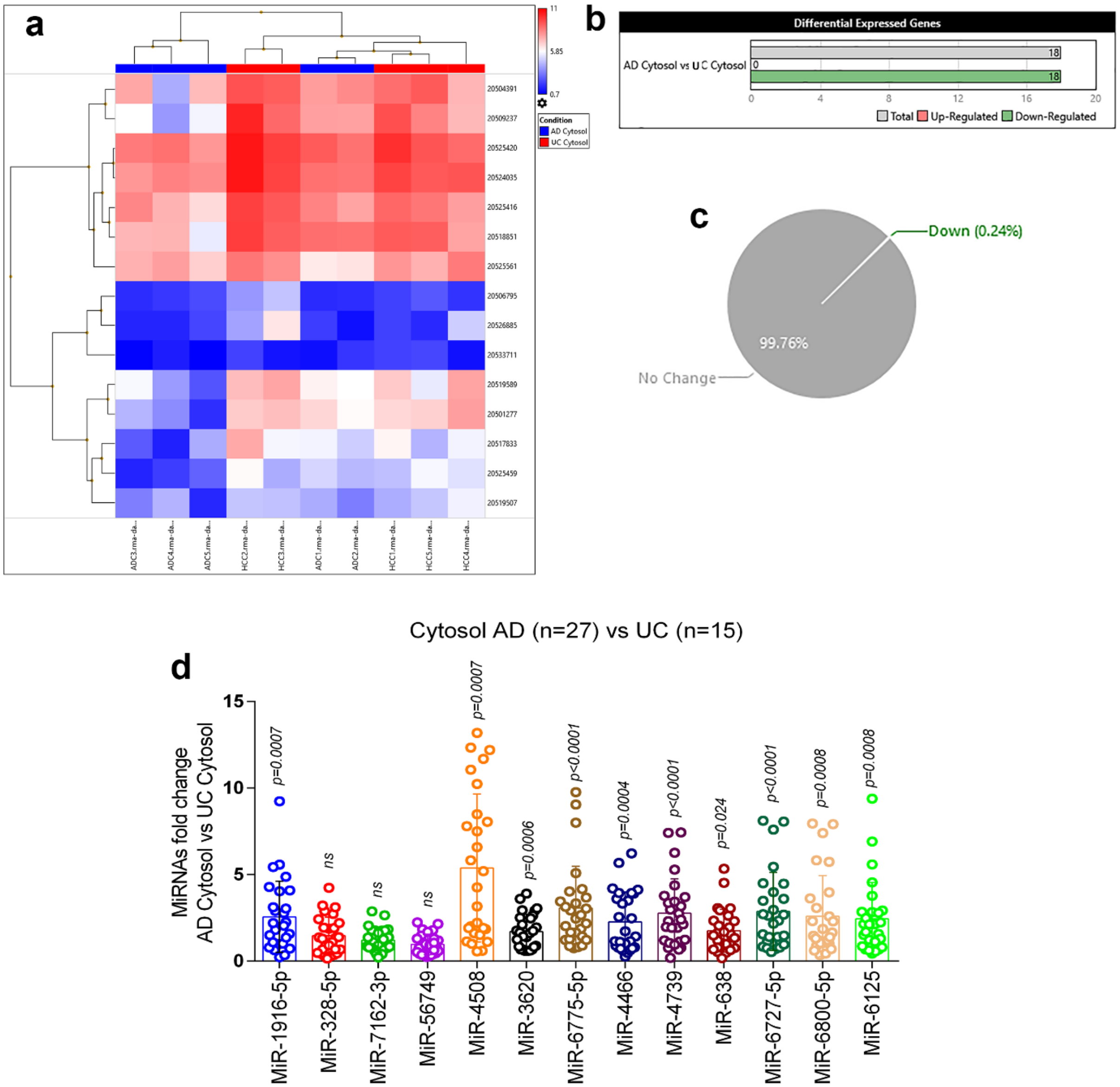

Supplementary Figure 4. (a) Hierarchical clustering and heat map of significantly deregulated miRNAs in AD cytosol and unaffected control cytosol. (b) Total number of miRNAs deregulated in cytosol in AD vs unaffected controls. (c) Percentage of miRNAs population changed in the cytosol in AD vs UC. (d) qRT-PCR based validation analysis of significantly deregulated miRNAs in unaffected control (n=15) and AD (n=27) cytosol. MiRNAs expression was quantified in AD vs UC cytosol. Values in the bar diagrams are mean ± SEM and error bars are equivalent throughout the figure (**d**).

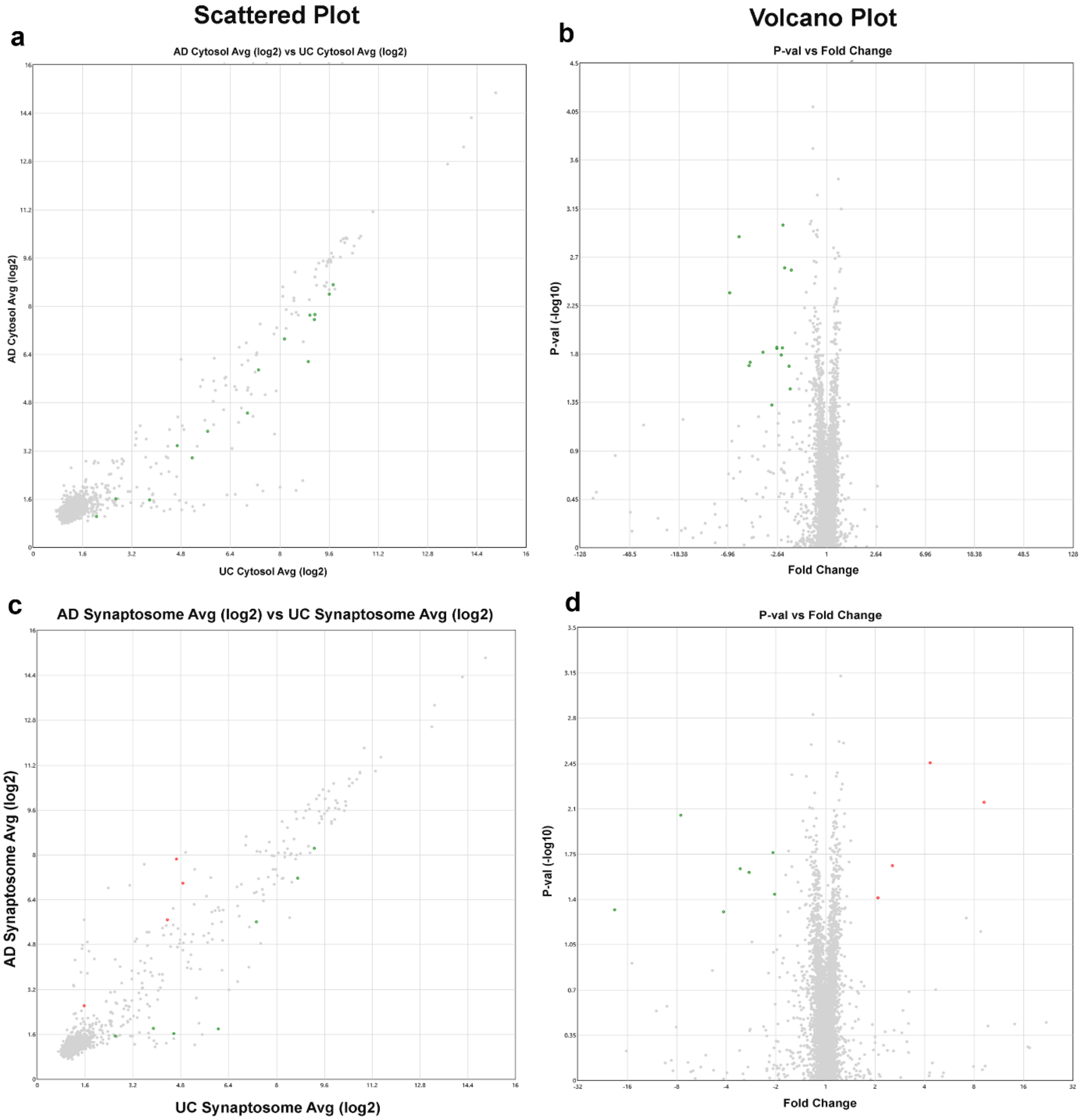

Supplementary Figure 5. (a) Scattered plot showing the average log2 fold changes values of significantly deregulated miRNAs in the cytosol in AD vs healthy state. (b) Volcano plot showing the p values (-log10) of significantly deregulated miRNAs in cytosol in AD vs healthy state. (c) Scattered plot showing the average log2 fold changes values of significantly deregulated miRNAs in the synaptosome in AD vs healthy state. (d) Volcano plot showing the p values (-log10) of significantly deregulated miRNAs in synaptosome in AD vs healthy state.

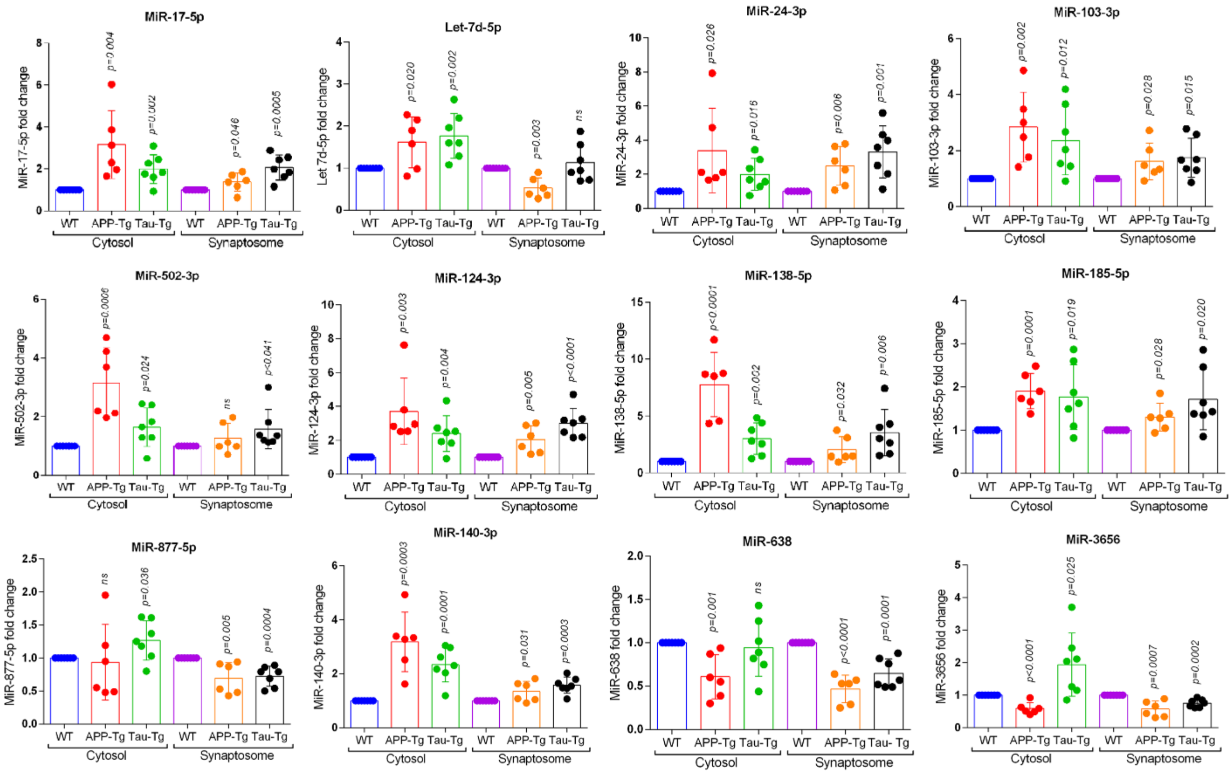

Supplementary Figure 6. Validation analysis of significantly deregulated cytosol miRNAs in WT mice (n=7), APP Tg mice (n=6) and Tau Tg mice (n=7). MiRNAs expression was quantified in WT vs APP Tg and Tau Tg mice in both cytosol and synaptosome. Values in the bar diagrams are mean ± SEM and error bars are equivalent throughout all the figures.

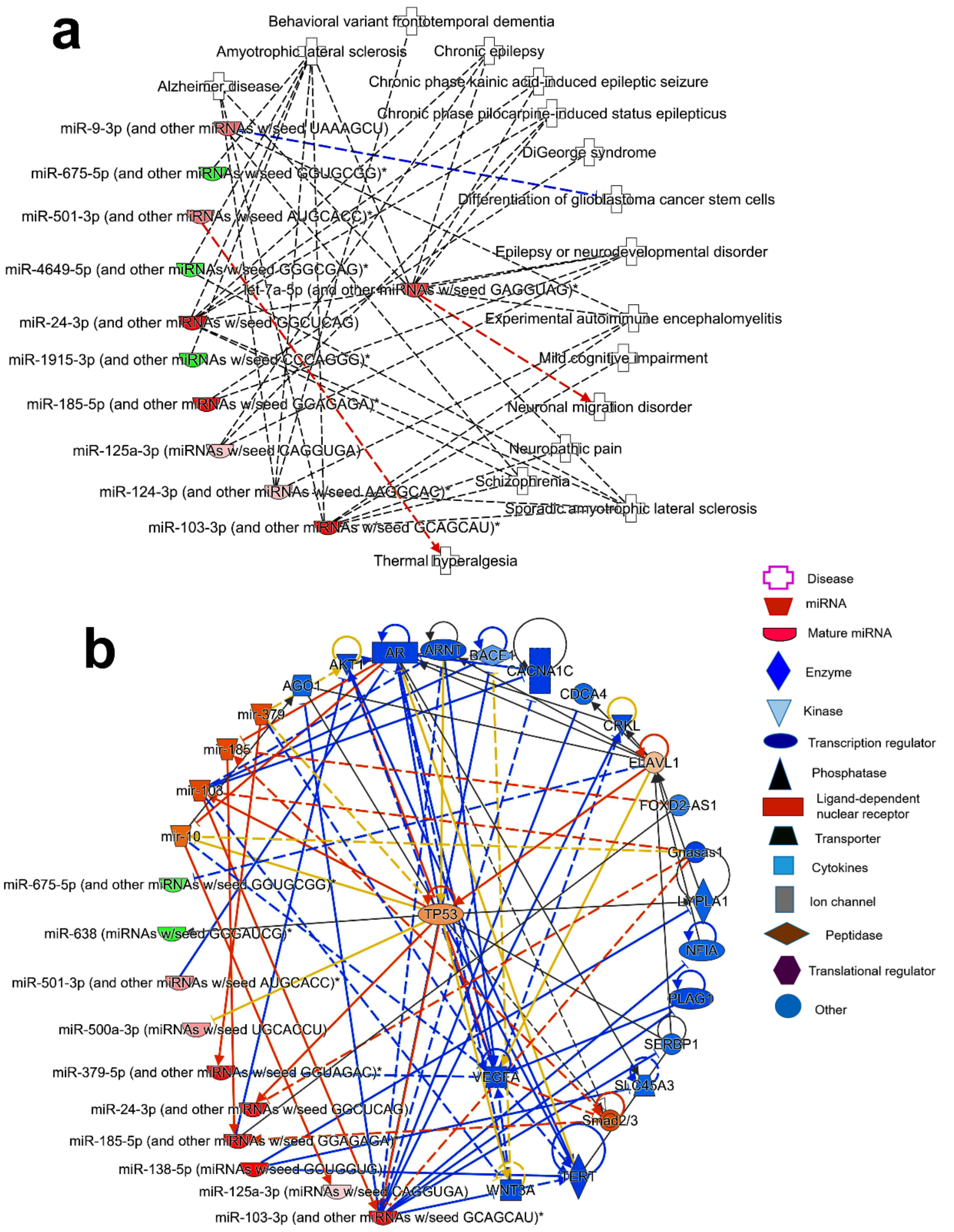

Supplementary Figure 7. Ingenuity Pathway Analysis of cytosolic and synaptosome miRNAs in healthy state (a) Healthy state- cytosolic and synaptosome miRNAs expression network in various human diseases. Green nodes represent decreased expression and red nodes represent increased expression of miRNAs. (b) MiRNAs target and seed sequences network of cytosolic and synaptosome miRNAs in the healthy state.

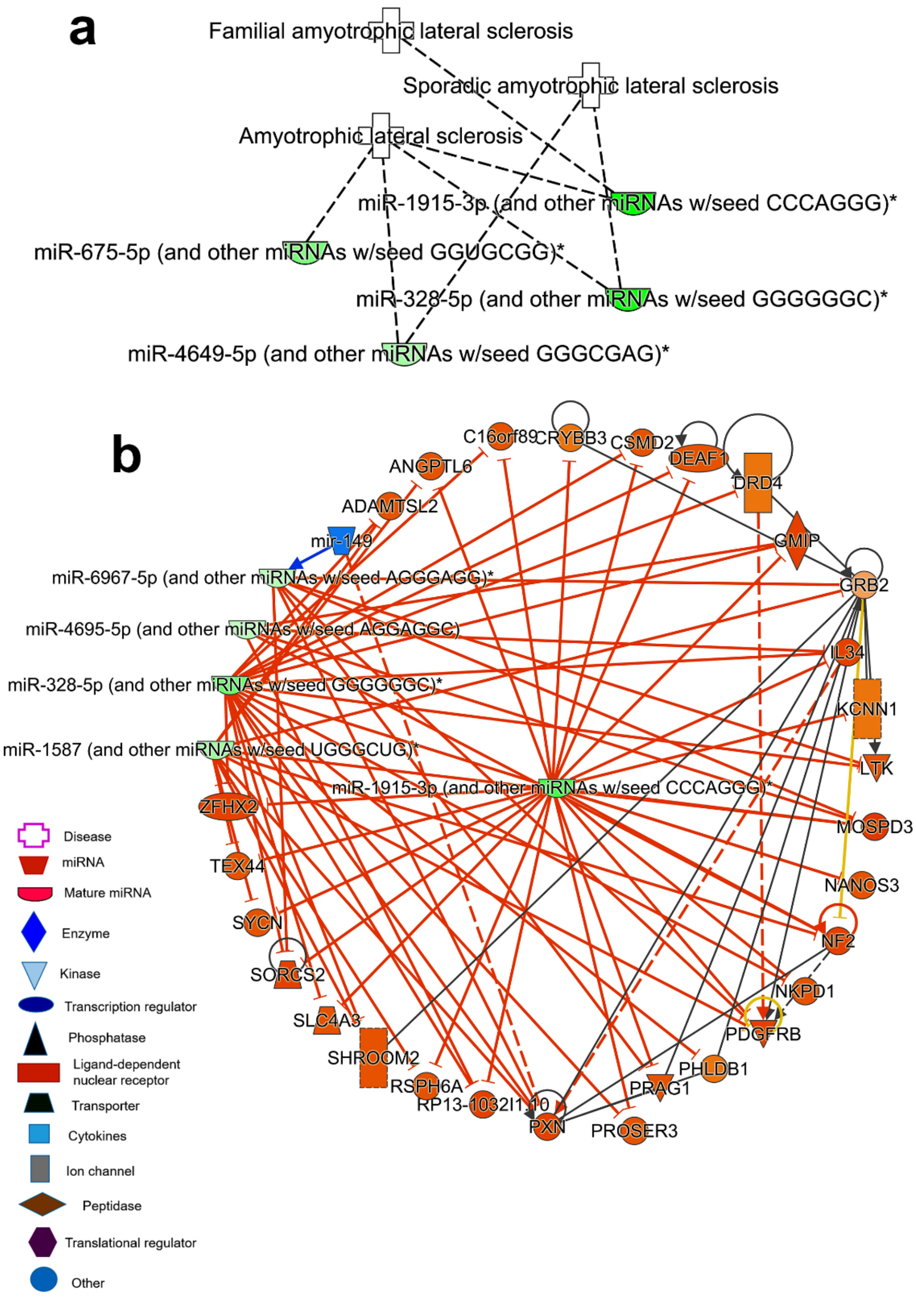

Supplementary Figure 8. Ingenuity Pathway Analysis of cytosolic miRNAs (a) Cytosolic miRNAs expression network in various human diseases. (b) Cytosolic miRNAs target and seed sequences network in AD and healthy state.

Homo sapiens gamma-aminobutyric acid type A receptor subunit alpha1 (GABRA1), transcript variant 1, mRNA

NCBI Reference Sequence: NM_000806.5

GenBank Graphics

>NM_000806.5 Homo sapiens gamma-aminobutyric acid type A receptor subunit alpha1 (GABRA1), transcript variant 1, mRNA

```
AACAGAAGATGCCGGAGAAGGGGGGGAAAAGTAGATGCGGATTTCGTCCTGACTTCTAAAAATTCCTCC
TCTCCCTCTCCCATTTTCCTAATCCGAGAATGATGGAGCTCGAGGCAAAGGAATGATTCCGGAAATGGAG
ATATGATTCTCAAACCTAGAAATGATCGGAGTGATTTATTAGTTAAATATTCTTCGTCCAGGAACCCAGC
ACAATTCAGAGCTGCAGATTGGATATTGGGAAGCAAATTTGGGTGTGAAATCTTCAGCAAAGGAGCACGC
AGAGTCCATGATGGCTCAGACCAAGTGAGTGAGAGGCAGAGCGAGGACGCCCCTCTGCTCTGGCGCGCCC
GGACTCGGACTCGCAGACTCGCGCTGGCTCCAGTCTCTCCACGATTCTCTCTCCCAGACTTTTCCCCGGT
CTTAAGAGATCCTGTGTCCAGAGGGGGCCTTAGCTGCTCCAGCCCGCGATGAGGAAAAGTCCAGGTCTGT
CTGACTGTCTTTGGGCCTGGATCCTCCTTCTGAGCACACTGACTGGAAGAAGCTATGGACAGCCGTCATT
ACAAGATGAACTTAAAGACAATACCACTGTCTTCACCAGGATTTTGGACAGACTCCTAGATGGTTATGAC
AATCGCCTGAGACCAGGATTGGGAGAGCGTGTAACCGAAGTGAAGACTGATATCTTCGTCACCAGTTTCG
GACCCGTTTCAGACCATGATATGGAATATACAATAGATGTATTTTTCCGTCAAAGCTGGAAGGATGAAAG
GTTAAAATTTAAAGGACCTATGACAGTCCTCCGGTTAAATAACCTAATGGCAAGTAAAATCTGGACTCCG
GACACATTTTTCCACAATGGAAAGAAGTCAGTGGCCCACAACATGACCATGCCCAACAAACTCCTGCGGA
TCACAGAGGATGGCACCTTGCTGTACACCATGAGGCTGACAGTGAGAGCTGAATGTCCGATGCATTTGGA
GGACTTCCCTATGGATGCCCATGCTTGCCCACTAAAATTTGGAAGTTATGCTTATACAAGAGCAGAAGTT
GTTTATGAATGGACCAGAGAGCCAGCACGCTCAGTGGTTGTAGCAGAAGATGGATCACGTCTAAACCAGT
ATGACCTTCTTGGACAAACAGTAGACTCTGGAATTGTCCAGTCAAGTACAGGAGAATATGTTGTTATGAC
CACTCATTTCCACTTGAAGAGAAAGATTGGCTACTTTGTTATTCAAACATACCTGCCATGCATAATGACA
GTGATTCTCTCACAAGTCTCCTTCTGGCTCAACAGAGAGTCTGTACCAGCAAGAACTGTCTTTGGAGTAA
CAACTGTGCTCACCATGACAACATTGAGCATCAGTGCCAGAAACTCCCTCCCTAAGGTGGCTTATGCAAC
AGCTATGGATTGGTTTATTGCCGTGTGCTATGCCTTTGTGTTCTCAGCTCTGATTGAGTTTGCCACAGTA
AACTATTTCACTAAGAGAGGTTATGCATGGGATGGCAAAAGTGTGGTTCCAGAAAAGCCAAAGAAAGTAA
AGGATCCTCTTATTAAGAAAAACAACACTTACGCTCCAACAGCAACCAGCTACACCCCTAATTTGGCCAG
GGGCGACCCGGGCTTAGCCACCATTGCTAAAAGTGCAACCATAGAACCTAAAGAGGTCAAGCCCGAAACA
AAACCACCAGAACCCAAGAAAACCTTTAACAGTGTCAGCAAAATTGACCGACTGTCAAGAATAGCCTTCC
CGCTGCTATTTGGAATCTTTAACTTAGTCTACTGGGCTACGTATTTAAACAGAGAGCCTCAGCTAAAAGC
CCCCACACCACATCAATAGATCTTTTACTCACATTCTGTTGTTCAGTCCTCTGCACTGGGAATTTATTTA
TGTTCTCAACGCAGTAATTCCCATCTGCTTTATTGCCTCTGTCTTAAAGAATTTGAAAGTTTCCTTATTT
TCATAATTCATTTAAGAACAAGAGACCCCTGTCTGGCAGTCTGGAGCAAAGCAGACTATGCAGCTTGGAG
ACAGGATTCTGACAGAGCAAGCGAAAGAGCAAAGTCATGTCAGAAGGAGACAGAATGAGAGAGAAAAGAG
GGGGAAGATGGTTCAAAGATACAAGAAAAAGTAGAAAAAAAAATAACACTTAACTAAAACCCCTAGGTCA
TTTGTAGATATATATTTCCAAATATTCTAAAAAAGATACTGTATATGTCAAAAATATTTTTATGTGAAGG
TGTTTCAAAGGGTAAATTATAAATGTTTCATGAAGAAAAAATTTTAAAAATCTACGTCTTTATTACACAA
ACTATGGTGTGCTTATGTTTTTGTTTTGCTTTTTAAACTGATGTATAGCTTTAACATTTTGTTTCCAAAG
CTGAAGATCCCCATTCTTTCTCTTTGAAAAAAAAAAAGGCCTAATGCATTATTTGTCATAAAATGCTAT
```

TTTAAAATTCATGGAACTTTCATACGTAAAGGTGCAGTTGCTCATTGTAGAGCACATTTAGTCCAATGAA
GATAAATGCTTTAAATAGTTTACTTCACTTTCATCTGAGCTTTTACCACTAGACTCAAGGAAGAATAATT
TTAACAGACATGTATACTCCATAGAAACTAAACTAAAATAGTTTAAAAATATTCCCTTTTTCACCCTATT
TTCAGATAGCACATGAGCCCAACACTCACTTAATTCTCATTATGAAGATGTTTTTAGAGGGGCAAAAATA
TTTTGCAAGCTCTGGAATTGTTGAATGTATTCTTTTATATAACTACATTAAAAGCTTTAGATTGAAATTT
ATGACTAGCAAACAAAAATAGAATATATAAACGATATATGTAAATATACAGCATGAGATTGTACATTTTT
TACTTTTTTAAAATTGTGTTCTTAAAATATTGTGTAAGAATCACTGCACTTAGCTGTTGGAATGTTGTTA
AATGCTATGGAAATACATTTAGAACCTGCATTTAAGAACAGAACAGCAAGTATGAACCACATGGAACTTA
AAACATATGGGTGTGAAGTCCACTTATGTAGACAAAACTTATAATTTCCAAACTGTTGTCTAGTATACAG
TGATCAGTTGCTCTCTGTTCAAGTCATTCCACACATTTCCCTATTTTAGGCTATTATAATATAGAAAGAA
AATGGGAAGCATTAGTTGGAGCTAGAAAATGAACTGTATATTATTGCTATATTTGCTAATACCAACTATT
TCAATAAGTGTTGTACCATATGTAGCATTAAATATAAAATACATAAAAGAATGTACAGAAAATAGCTTTT
ATTGAGTAATATTACATTTCATTTATACTGTAGCAATATATTTGTAGGTATACTATGTAAGGGCTTTAAA
TAAAAGAGGTCCATTAATACTTCCTTATAAAAATTCTAGTCTGTTTCATTACTGCCCAGATGTTTTAGAG
ATAAATATTTATGCAGAAGGTATTTTTGAAGTCTCCTTTTGTCTGATAGAGTTTAACAGATATTTAAATT
TAGTGCTCAGAATCCACAAGTCACGGTCTAAACACACTTAGAATACTACAGCATAAATCTGTTAGCATTA
TTGCCAAATAAGACAGTTGGGATCCAAACCCAAGTCTTGAGCAATGTTTTTCTCAAAAAGCTGCTATCCA
ATGATATAGGAAAATACATTGTGTTTTCCTAAACACACTTTTCTTTTTAAATGTGCTTCATTGTTTGATT
TGGTCCTGCCTAAATTTCACAAGCTAGGCCAATGAAGGCTGAATCAAAGACATTTCATCCACCAATATCA
TGTGTAGATATTATGTATAGAAAATAAAATAAATTATGGCTCTAACTTCTGTGTTGCTGTTTATCTTGTT
ATTTTTCGGCGTTATACTAATGTGTTTATTGAGAGCATTTTACCTTCCAGACTTCTCATGGCTAACTTTT
GGTCTGTATTTTGCTCCTTAGATGTGAATATTTCTTATTAGTCTGCTTCCTGCTACGCAATGACTGCATT
TCTATCATTTCTCAGTTTGTTAGTATATGTGGATAGTATTCTACTGTATAAATGATTGCAAAGTTTATCA
AAAACAAATTATTATATGTAGCTTTTCTACAGTGCTTTGCTAAACCATGTAGTACTAGTTAAGTCTTCCT
TGAAAATAAAGATACACTCTTATAGGGGACAGTTCCTGTTCACTCCCAGGAAACTTTTTTAAAAGATGAC
ACTGAATGTTTATTGCACTTTAGTGCAGTGAAGTGGCAATAAAACCTAACATGAATCAAGGTTGTTTATG
GCAGATGCATGTGTTGCTTTACAGAGTTTAGCAAAAGCTCTTAATTTTATGTCATACTGTATTCTACTGA
ATAATAAAGCTAACATTATTCAATAATAAAATGGAA

Supplementary Figure 9. NCBI mRNA target analysis of miR-501-3p and miR-502-3p showed the five seed sequences binding site of both miRNAs with gamma-aminobutyric acid type A receptor subunit alpha1 gene. The seed sequence of both miRNAs is similar.

Gene Ontology Enrichment Analysis by ShinyGO v0.61

Hsa-miR-502-3p

↓

Validated and predicted targets (n=461) by TAC software

↓

ShinyGO v0.61

Hsa-miR-502-3p- GO Biological Process

Enrichment FDR	Genes in list	Total genes	Functional Category
0.009807	126	2561	Response to external stimulus
0.044273	116	2474	Nervous system development
0.044273	129	2785	Anatomical structure morphogenesis
0.044273	157	3529	Regulation of signal transduction
0.044273	170	3903	Regulation of cell communication
0.044467	171	3952	Regulation of signaling

Hsa-miR-502-3p- GO cellular component

Enrichment FDR	Genes in list	Total genes	Functional Category
0.028981	11	79	GABA-ergic synapse
0.045359	65	1269	Microtubule cytoskeleton
0.045359	16	178	Growth cone
0.045359	16	181	Site of polarized growth

Hsa-miR-502-3p- GO Molecular function

Enrichment FDR	Genes in list	Total genes	Functional Category
0.02048	19	206	Calmodulin binding
0.02048	7	32	WW domain binding
0.033217	9	61	Oxidoreductase activity, acting on peroxide as acceptor
0.047728	4	11	Lysozyme activity
0.047728	8	56	Peroxidase activity
0.047728	11	99	Antioxidant activity

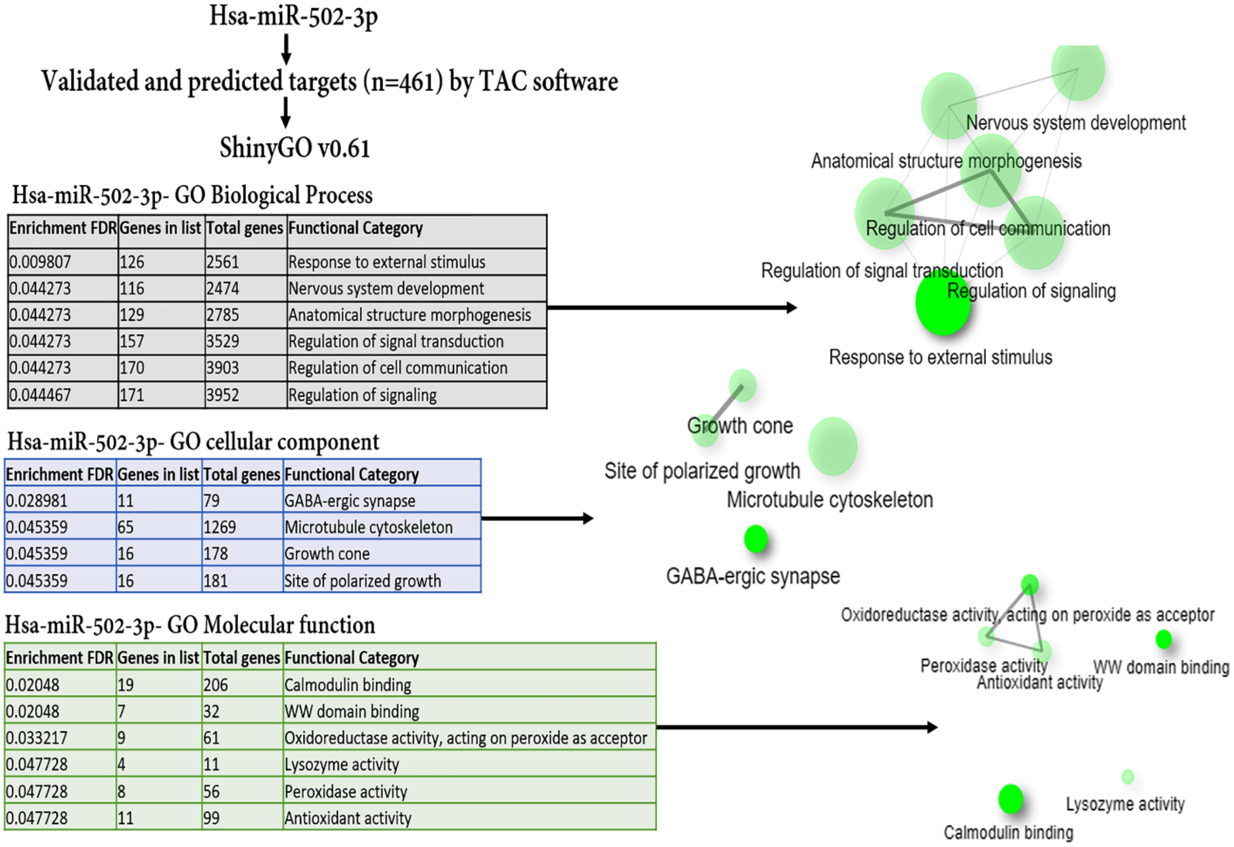

Supplementary Figure 10. Gene Ontology Enrichment analysis of miR-502-3p. Network shows the relationship between enriched terms provided by the ShinyGO v0.61 platform. MiR-502-3p significantly involved in several biological process, cellular components and molecular functions. Darkness of nodes coded with the level of significance of the enrichment, while node size is representative for the gene set size.

Supplementary Table 1- Details of human postmortem brains

1. Human Brains and Spinal Fluid Resource center, Los Angeles							
**S. No**	**HSB#**	**Age**	**Sex**	**Neuropathology**	**Coronal slab#**	**Structure**	**Autolysis time (hr)**
1	4513	74	M	AD	1	Broadmann's Area 10	15.6
2	4498	76	M	AD	1	Broadmann's Area 10	12.9
3	4204	68	M	AD	1	Broadmann's Area 10	11.9
4	4203	72	F	AD	1	Broadmann's Area 10	20.3
5	4454	82	F	AD	1	Broadmann's Area 10	9
6	4043	80	F	AD	1	Broadmann's Area 10	13
7	4382	74	F	AD	1	Broadmann's Area 10	16.2
8	4617	73	F	AD	1	Broadmann's Area 10	18.9
9	4718	93	F	AD	1	Broadmann's Area 10	8.2
10	4608	80	M	AD	1	Broadmann's Area 10	3.1
11	4752	89	M	AD	1	Broadmann's Area 10	9
12	4788	65	M	AD	1	Broadmann's Area 10	7.8
13	4130	67	F	Normal	1	Broadmann's Area 10	11.8
14	4431	68	F	Normal	1	Broadmann's Area 10	23.7
15	4660	73	F	Normal	1	Broadmann's Area 10	18.5
16	5072	83	M	Normal	1	Broadmann's Area 10	19.5
17	5190	68	M	Normal	1	Broadmann's Area 10	20.3
**2. Brain Endowment Bank University of Miami**							
**S.No.**	**Tissue code**	**Age**	**Sex**	**Brain type**	**Race**	**Structure**	**Autolysis time**
1	HBFR1703	69	F	AD	C	Broadmann's Area 10	22
2	HBFQ1711	77	M	AD	C	Broadmann's Area 10	18

3	HBJG1710	79	M	AD	C	Broadmann's Area 10	23.8
4	HBDA1704	80	M	AD	C	Broadmann's Area 10	22.1
5	HCTYN1713	80	F	AD	C	Broadmann's Area 10	6.5
6	HBDI1710	85	F	AD	C	Broadmann's Area 10	8
7	HBEM1701	86	M	AD	C	Broadmann's Area 10	15.5
8	HBIP1701	90	F	AD	C	Broadmann's Area 10	22.1
9	HBCG1703	90	F	AD	C	Broadmann's Area 10	8.5
10	HCTZX1702	95	M	AD	C	Broadmann's Area 10	19.8
11	HCT15HAO1713	70	M	Control	C	Broadmann's Area 10	12.7
12	HCTZZC1711	82	F	Control	C	Broadmann's Area 10	14.2
13	HCT15HBC1709	83	M	Control	C	Broadmann's Area 10	25
14	HCTZZT1702	84	M	Control	C	Broadmann's Area 10	15.5
15	HCT15HBU1704	91	F	Control	C	Broadmann's Area 10	18.7
**3. Mount Sinai NIH Brain and Tissue Repository**							
**S. No.**	**Barcode**	**Age**	**Sex**	**DX**	**Race**	**Brain region**	**PMI**
1	77423	79	F	AD	W	Broadmann's Area 10	6.50
2	77424	69	M	AD	W	Broadmann's Area 10	5.42
3	77425	75	M	AD	W	Broadmann's Area 10	8.00
4	77426	94	F	AD	W	Broadmann's Area 10	4.33
5	77427	82	M	AD	W	Broadmann's Area 10	20.67
6	77428	65	M	NL	H	Broadmann's Area 10	3.83
7	77431	103	F	NL	W	Broadmann's Area 10	3.83
8	77433	75	M	NL	B	Broadmann's Area 10	5.00

9	77436	93	M	NL	W	Broadmann's Area 10	4.17
10	77437	84	F	NL	W	Broadmann's Area 10	5.48

Supplementary Table 2. Summary of antibody dilutions and conditions used in the immunoblotting analysis of synaptosomes

Marker	Primary Antibody – Species and Dilution	Purchased from Company, City & State	Secondary Antibody, Dilution	Purchased from Company, City & State
PSD95	Rabbit Monoclonal 1:300	Novus Biological, Littleton, CO	Donkey Anti-rabbit HRP 1:10,000	GE Healthcare Amersham, Piscataway, NJ
SNAP25	Rabbit Polyclonal 1:500	Novus Biological, Littleton, CO	Donkey Anti-rabbit HRO 1:10,000	GE Healthcare Amersham, Piscataway, NJ
SYN	Rabbit Polyclonal 1:500	Novus Biological, Littleton, CO	Donkey Anti-rabbit HRP 1:10,000	GE Healthcare Amersham, Piscataway, NJ
PCNA	Mouse Monoclonal 1:500	Santa Cruz Biotechnology, Inc., Dallas, TX	Sheep Anti-mouse HRP 1:10,000	GE Healthcare Amersham, Piscataway, NJ
elF2a	Mouse Monoclonal 1:500	Cell Signaling Technology, Danvers, MA	Sheep Anti-mouse HRP 1:10,000	GE Healthcare Amersham, Piscataway, NJ
GABAR1a	Rabbit Polyclonal 1:500	Bioss Antibodies, Woburn, MA	Donkey Anti-rabbit HRP 1:10,000	GE Healthcare Amersham, Piscataway, NJ
VGLUT1	Rabbit Polyclonal 1:500	Thermo Fisher Scientific, Waltham, MA	Donkey Anti-rabbit HRP 1:10,000	GE Healthcare Amersham, Piscataway, NJ
NueN	Rabbit Monoclonal 1:1000	Abcam, Cambridge, UK	Donkey Anti-rabbit HRP 1:10,000	GE Healthcare Amersham, Piscataway, NJ

IBa1/AIF-1	Rabbit Monoclonal 1:1000	Cell Signaling Technology, Danvers, MA	Donkey Anti-rabbit HRP 1:10,000	GE Healthcare Amersham, Piscataway, NJ
AGO2	Rabbit Monoclonal 1:500	Cell Signaling Technology, Danvers, MA	Donkey Anti-rabbit HRP 1:10,000	GE Healthcare Amersham, Piscataway, NJ
Drosha	Rabbit Monoclonal 1:500	Cell Signaling Technology, Danvers, MA	Donkey Anti-rabbit HRP 1:10,000	GE Healthcare Amersham, Piscataway, NJ
Dicer	Rabbit Polyclonal 1:500	Thermo Fisher Scientific, Waltham, MA	Donkey Anti-rabbit HRP 1:10,000	GE Healthcare Amersham, Piscataway, NJ
6E10	Mouse Monoclonal 1:500	Biolegend, San Diego, CA	Sheep Anti-mouse HRP 1:10,000	GE Healthcare Amersham, Piscataway, NJ
p-Tau	Mouse Monoclonal 1:500	Thermo Fisher Scientific, Waltham, MA	Sheep anti-mouse HRP 1:10 000	GE Healthcare Amersham, Piscataway, NJ
B-actin	Mouse Monoclonal 1:500	Sigma-Aldrich St Luis, MO	Sheep anti-mouse HR 1:10,000	GE Healthcare Amersham Piscataway, NJ

Supplementary Table 3. List of selected miRNAs and forward primer sequence

S. No.	MiRNA name	Forward Primer Sequences (5'-3')
1	miR-124-3p	Forward primer- TAAGGCACGCGGTGAATGCCAA
2	let-7a-5p	Forward primer-TGAGGTAGTAGGTTGTATAGTT
3	MiR-103a-3p	Forward primer- AGCAGCATTGTACAGGGCTATGA
4	miR-24-3p	Forward primer- TGGCTCAGTTCAGCAGGAACAG
5	miR-151a-5p	Forward primer- TCGAGGAGCTCACAGTCTAGT
6	miR-185-5p	Forward primer- TGGAGAGAAAGGCAGTTCCTGA
7	miR-140-3p	Forward primer- TACCACAGGGTAGAACCACGG
8	let-7d-5p	Forward primer- AGAGGTAGTAGGTTGCATAGTT
9	miR-138-5p	Forward primer- AGCTGGTGTTGTGAATCAGGCCG
10	MiR-4284	Forward primer- GGGCTCACATCACCCCAT
11	MiR-320a-3p	Forward primer- AAAAGCTGGGTTGAGAGGGCGA
12	MiR-485-5p	Forward primer- AGAGGCTGGCCGTGATGAATTC
13	let-7e-5p	Forward primer- TGAGGTAGGAGGTTGTATAGTT
14	miR-502-3p	Forward primer- AATGCACCTGGGCAAGGATTCA
15	miR-500a-3p	Forward primer- ATGCACCTGGGCAAGGATTCTG
16	miR-17-5p	Forward primer- CAAAGTGCTTACAGTGCAGGTAG
17	miR-379-5p	Forward primer- TGGTAGACTATGGAACGTAGG
18	miR-4485-3p	Forward primer- TAACGGCCGCGGTACCCTAA
19	miR-491-5p	Forward primer- AGTGGGGAACCCTTCCATGAGG
20	miR-320b	Forward primer- AAAAGCTGGGTTGAGAGGGCAA
21	miR-320c	Forward primer- AAAAGCTGGGTTGAGAGGGT
22	miR-331-3p	Forward primer- GCCCCTGGGCCTATCCTAGAA
23	miR-501-3p	Forward primer- AATGCACCCGGGCAAGGATTCT
24	miR-106a-5p	Forward primer- AAAAGTGCTTACAGTGCAGGTAG
25	let-7c-5p	Forward primer- TGAGGTAGTAGGTTGTATGGTT
26	miR-6750-5p	Forward primer- CAGGGAACAGCTGGGTGAGCTGCT
27	let-7f-5p	Forward primer- TGAGGTAGTAGATTGTATAGTT
28	miR-9-3p	Forward primer- ATAAAGCTAGATAACCGAAAGT
29	miR-5787	Forward primer- GGGCTGGGGCGCGGGGAGGT
30	miR-3196	Forward primer- CGGGGCGGCAGGGGCCTC
31	miR-3656	Forward primer- GGCGGGTGCGGGGGTGG
32	miR-6724-5p	Forward primer- CTGGGCCCGCGGCGGGCGTGGGG
33	miR-4516	Forward primer- GGGAGAAGGGTCGGGGC
34	miR-762	Forward primer- GGGGCTGGGGCCGGGGCCGAGC
35	miR-8069	Forward primer- GGATGGTTGGGGGCGGTCGGCGT
36	miR-6821-5p	Forward primer- GTGCGTGGTGGCTCGAGGCGGGG
37	miR-4433b-3p	Forward primer- CAGGAGTGGGGGGTGGGACGT

38	miR-7162-3p	Forward primer- TCTGAGGTGGAACAGCAGC
39	miR-6800-5p	Forward primer- GTAGGTGACAGTCAGGGGCGG
40	miR-4270	Forward primer- TCAGGGAGTCAGGGGAGGGC
41	miR-4459	Forward primer- CCAGGAGGCGGAGGAGGTGGAG
42	miR-6775-5p	Forward primer- TCGGGGCATGGGGGAGGGAGGCTGG
43	miR-4695-5p	Forward primer- CAGGAGGCAGTGGGCGAGCAGG
44	miR-638	Forward primer- AGGGATCGCGGGCGGGTGGCGGCCT
45	miR-4687-3p	Forward primer- TGGCTGTTGGAGGGGGCAGGC
46	miR-1915-3p	Forward primer- CCCCAGGGCGACGCGGCGGG
47	miR-6869-5p	Forward primer- GTGAGTAGTGGCGCGCGGCGGC
48	miR-4466	Forward primer- GGGTGCGGGCCGGCGGGG
49	miR-2861	Forward primer- GGGGCCTGGCGGTGGGCGG
50	miR-4488	Forward primer- AGGGGGCGGGCTCCGGCG
51	miR-6729-5p	Forward primer- TGGGCGAGGGCGGCTGAGCGGC
52	miR-328-5p	Forward primer- GGGGGGGCAGGAGGGGCTCAGGG
53	miR6749-5p	Forward primer- TCGGGCCTGGGGTTGGGGGAGC
54	miR-3620-5p	Forward primer- GTGGGCTGGGCTGGGCTGGGCC
55	miR-4739	Forward primer- AAGGGAGGAGGAGCGGAGGGGCCCT
56	miR-6727-5p	Forward primer- CTCGGGGCAGGCGGCTGGGAGCG
57	miR-6125	Forward primer- GCGGAAGGCGGAGCGGCGGA
58	miR-1202	Forward primer- GTGCCAGCTGCAGTGGGGGAG
59	miR-877-5p	Forward primer- GTAGAGGAGATGGCGCAGGG
60	miR-664b-3p	Forward primer- TTCATTTGCCTCCCAGCCTACA
61	miR-6511b-5p	Forward primer- CTGCAGGCAGAAGTGGGGCTGACA
62	miR-4508	Forward primer- GCGGGGCTGGGCGCGCG
63	miR-1237-5p	Forward primer- CGGGGGCGGGGCCGAAGCGCG
64	miR-5001-5p	Forward primer- AGGGCTGGACTCAGCGGCGGAGCT
65	miR-4492	Forward primer- GGGGCTGGGCGCGCGCC
66	miR-4497	Forward primer- CTCCGGGACGGCTGGGC
67	miR-107	Forward primer- AGCAGCATTGTACAGGGCTATCA
69	miR-6821-5p	Forward primer- GTGCGTGGTGGCTCGAGGCGGGG
71	miR-7847-3p	Forward primer- CGTGGAGGACGAGGAGGAGGC
73	miR-6085	Forward primer- AAGGGGCTGGGGGAGCACA
75	miR-1469	Forward primer- CTCGGCGCGGGGCGCGGGCTCC
77	miR-6786-5p	Forward primer- GCGGTGGGGCCGGAGGGGCGT
79	miR-7110-5p	Forward primer- TGGGGGTGTGGGGAGAGAGAG
81	miR-4516	Forward primer- GGGAGAAGGGTCGGGGC
83	miR-1207-5p	Forward primer- TGGCAGGGAGGCTGGGAGGGG
84	U6 SnRNA	Forward primer- CGCTTCGGCAGCACATATACTAA<br>Reverse Primer- TATGGAACGCTTCACGAATTTGC

85	Sno-202	Forward primer- AGTACTTTTGAACCCTTTTCCA
86	PSD95	Forward primer- CTTCATCCTTGCTGGGGGTC<br>Reverse primer - TTGCGGAGGTCAACACCATT
87	SNAP25	Reverse primer - GTGGTCATCTGGTGGCTCTA<br>Reverse primer- TTCAGCAAACGCCACTGAGA
88	SYN	Forward primer- CTGCGTTAAAGGGGGCACTA<br>Reverse primer- ACAGCCACGGTGACAAAGAA
89	PCNA	Forward primer- AGGGTTGGTAGTTGTCGCTG<br>Reverse primer - CAAACATGGTGGCGGAGTTG
98	elF1a	Forward primer- AGGATGGGACCTTGTTTGCC<br>Reverse primer - AAACTGCCTTCAGGAGGTCG
90	B-actin	Forward primer- AGAAGCTGTGCTATGTTGCTCTA<br>Reverse primer- TCAGGCAGCTCATAGCTCTTC

Supplementary Table 4. Details of significantly deregulated miRNA candidates in unaffected controls synaptosomes vs cytosol

ID	Transcript ID(Array Design)	HC Synaptosome Avg (log2)	HC Cytosol Avg (log2)	HC Synaptosome Standard Deviation	HC Cytosol Standard Deviation	Fold Change	HC Synaptosome Expressed	HC Cytosol Expressed	Expressed in Both Conditions	P-val	Accession	Chromosome	Strand	Start	Stop
20500191	hsa-miR-103a-3p	10.46	2.55	2.51	3.42	239.8	T	T	T	0.0099	MIMAT0000101	chr20	+	3898188	3898210
20500196	hsa-miR-107	9.26	2.02	3.24	3.37	151.67	T	T	T	0.0413	MIMAT0000104	chr10	-	91352513	91352535
20500787	hsa-miR-185-5p	8.58	1.41	1.49	1.89	144.25	T	F	F	0.0003	MIMAT0000455	chr22	+	20020676	20020697
20500743	hsa-miR-138-5p	7.89	1.28	3.48	2.46	97.77	T	F	F	0.0424	MIMAT0000430	chr16	+	56892439	56892461
20518879	hsa-miR-4485	7.67	1.24	1.31	2.25	86.21	T	F	F	0.0009	MIMAT0019019	chr11	-	10529824	10529843
20500713	hsa-let-7g-5p	6.63	1.51	3.04	0.76	34.89	T	F	F	0.0492	MIMAT0000414	chr3	-	52302352	52302373

20500148	hsa-miR-24-3p	9.1	4.5	1.2	2.44	24.29	T	T	T	0.0013	MIMAT0000080	chr19	-	13947103	13947124
20517744	hsa-miR-4284	6.18	1.72	1.6	0.37	22	T	F	F	0.0002	MIMAT0016915	chr7	+	73125657	73125674
20500123	hsa-let-7f-5p	5.52	1.52	3.18	0.25	16.06	T	T	T	0.0296	MIMAT0000067	chr9	+	96938635	96938656
20500764	hsa-miR-9-3p	4.86	1.36	2.1	0.23	11.33	T	F	F	0.028	MIMAT0000442	chr1	-	1.56E+08	1.56E+08
20503876	hsa-miR-500a-3p	4.83	1.52	1.29	0.22	9.93	T	F	F	0.0011	MIMAT0002871	chrX	+	49773090	49773111
20500121	hsa-let-7e-5p	11.4	8.19	1.43	1.69	9.26	T	T	T	0.0082	MIMAT0000066	chr19	+	52196046	52196067
20503880	hsa-miR-502-3p	4.75	1.59	0.76	0.39	8.96	T	F	F	1.15E-05	MIMAT0004775	chrX	+	49779257	49779278
20504562	hsa-miR-320b	9.76	7.19	0.68	1.64	5.96	T	T	T	0.0061	MIMAT0005792	chr1	+	1.17E+08	1.17E+08
20500848	hsa-mi	9.95	7.49	0.56	1.71	5.51	T	T	T	0.0055	MIMAT0000510	chr8	-	22102488	22102509

	R-320a														
20500112	hsa-let-7a-5p	10.6	8.29	2.14	2.15	4.97	T	T	T	0.0465	MIMAT0000062	chr11	-	1.22E+08	1.22E+08
20500119	hsa-let-7d-5p	10.47	8.18	1.83	0.93	4.91	T	T	T	0.0158	MIMAT0000065	chr9	+	96941123	96941144
20500729	hsa-miR-124-3p	10.95	8.84	1.41	3.27	4.32	T	T	T	0.0062	MIMAT0000422	chr20	+	61809904	61809923
20504563	hsa-miR-320c	9.32	7.28	0.48	1.2	4.11	T	T	T	0.0032	MIMAT0005793	chr18	+	19263520	19263539
20500117	hsa-let-7c-5p	11.63	9.86	1.62	0.66	3.39	T	T	T	0.0128	MIMAT0000064	chr21	+	17912158	17912179
20534575	hsa-mir-124-1	10.66	8.94	1.36	3.26	3.28	T	T	T	0.0143	MI0000443	chr8	-	9760898	9760982
20534578	hsa-mir-124-2	10.66	8.94	1.36	3.26	3.28	T	T	T	0.0143	MI0000444	chr8	+	65291706	65291814
20534580	hsa-mir-124-3	10.66	8.94	1.36	3.26	3.28	T	T	T	0.0143	MI0000445	chr20	+	61809852	61809938

20518913	hsa-miR-4516	8.46	9.46	0.68	0.46	-2.01	T	T	T	0.0054	MIMAT0019053	chr16	+	2183121	2183137
20525420	hsa-miR-6729-5p	9.02	10.04	0.53	0.67	-2.02	T	T	T	0.0079	MIMAT0027359	chr1	+	12089220	12089241
20526178	hsa-miR-7110-5p	1.58	2.65	0.39	0.91	-2.09	T	T	T	0.0384	MIMAT0028117	chr3	+	1.23E+08	1.23E+08
20514163	hsa-miR-2861	7.65	8.79	0.8	0.27	-2.2	T	T	T	0.0093	MIMAT0013802	chr9	+	1.31E+08	1.31E+08
20525533	hsa-miR-6786-5p	7.32	8.48	1.06	0.65	-2.23	T	T	T	0.0239	MIMAT0027472	chr17	+	79660839	79660859
20518882	hsa-miR-4488	8.4	9.57	0.55	0.41	-2.24	T	T	T	0.0123	MIMAT0019022	chr11	+	61276071	61276088
20518851	hsa-miR-4466	8.2	9.36	1.63	0.81	-2.25	T	T	T	0.0399	MIMAT0018993	chr6	-	1.57E+08	1.57E+08
20506801	hsa-miR-120	5.56	6.74	1.19	0.74	-2.27	T	T	T	0.0105	MIMAT0005871	chr8	+	1.29E+08	1.29E+08

	7-5p														
20537543	hsa-mir-6800	6.07	7.26	0.49	0.82	-2.27	T	T	T	0.0113	MI0022645	chr19	+	50335275	50335356
20537544	hsa-mir-6800	6.12	7.35	0.48	0.91	-2.33	T	T	T	0.0129	MI0022645	chr19	+	50335275	50335356
20507742	hsa-miR-1469	6.27	7.52	0.89	0.93	-2.38	T	T	T	0.0289	MIMAT0007347	chr15	+	96876490	96876511
20517907	hsa-miR-3656	7.5	8.88	0.37	0.63	-2.61	T	T	T	0.0024	MIMAT0018076	chr11	+	1.19E+08	1.19E+08
20509237	hsa-miR-1915-3p	7.78	9.21	1.96	1.17	-2.7	T	T	T	0.0499	MIMAT0007892	chr10	-	21785505	21785524
20504391	hsa-miR-638	7.94	9.41	0.81	0.82	-2.77	T	T	T	0.0138	MIMAT0003308	chr19	+	10829095	10829119
20519493	hsa-miR-4687-3p	7.8	9.28	0.69	0.27	-2.78	T	T	T	0.0009	MIMAT0019775	chr11	+	3877343	3877363
20525511	hsa-miR-677	5.89	7.48	0.99	1.08	-3.03	T	T	T	0.036	MIMAT0027450	chr16	-	87868237	87868261

	5-5p														
20525396	hsa-miR-6724-5p	7.16	8.87	0.89	0.28	-3.26	T	T	T	0.0006	MIMAT0025856				
20518839	hsa-miR-4459	3.79	5.6	1.3	1.17	-3.51	T	T	T	0.012	MIMAT0018981	chr5	-	53371390	53371411
20525561	hsa-miR-6800-5p	6.58	8.51	0.75	0.54	-3.81	T	T	T	0.0018	MIMAT0027500	chr19	+	50335279	50335299
20517729	hsa-miR-4270	4.79	6.79	1.5	0.38	-4.01	T	T	T	0.0059	MIMAT0016900	chr3	-	15537786	15537805
20523017	hsa-miR-6085	4.2	6.3	1.83	0.72	-4.31	T	T	T	0.0309	MIMAT0023710	chr15	+	62635310	62635328
20529131	hsa-miR-4433b-3p	3.5	5.65	1.43	0.46	-4.45	T	T	T	0.0042	MIMAT0030414	chr2	-	64567903	64567923
20519507	hsa-miR-4695-5p	3.48	5.63	0.97	0.61	-4.46	T	T	T	0.0009	MIMAT0019788	chr1	-	19209744	19209765

20529139	hsa-miR-7847-3p	4.78	7.37	1.92	1.29	-6.02	T	T	T	0.0359	MIMAT0030422	chr11	+	1901335	1901355
20525603	hsa-miR-6821-5p	3.87	6.9	1.64	1.76	-8.17	T	T	T	0.0494	MIMAT0027542	chr22	+	50356514	50356536

Supplementary Table 5. Details of significantly deregulated miRNA candidates in AD synaptosomes vs cytosol

ID	Transcript ID(Array Design)	AD Synaptosome Avg (log2)	AD Cytosol Avg (log2)	AD Synaptosome Standard Deviation	AD Cytosol Standard Deviation	Fold Change	P-val	AD Synaptosome Expressed	AD Cytosol Expressed	Expressed in Both Conditions	Accession	Chromosome	Strand	Start	Stop
20500729	hsa-miR-124-3p	10.45	1.95	0.8	3.67	361.92	0.0018	T	T	T	MIMAT0000422	chr20	+	61809904	61809923
20500112	hsa-let-7a-5p	10.23	2.07	2.4	3.55	284.64	0.0096	T	T	T	MIMAT0000062	chr11	-	1.22E+08	1.22E+08
20534575	hsa-mir-124-1	10.03	2.17	0.77	3.39	233.1	0.002	T	T	T	MI0000443	chr8	-	9760898	9760982
20534578	hsa-mir-124-2	10.03	2.17	0.77	3.39	233.1	0.002	T	T	T	MI0000444	chr8	+	65291706	65291814
20534580	hsa-mir-124-3	10.03	2.17	0.77	3.39	233.1	0.002	T	T	T	MI0000445	chr20	+	61809852	61809938
20500148	hsa-miR-24-3p	9.09	1.25	0.57	2.95	229.12	0.0007	T	F	F	MIMAT0000080	chr19	-	13947103	13947124
20500787	hsa-miR-185-5p	7.87	1.04	2.82	1.75	113.71	0.0071	T	F	F	MIMAT0000455	chr22	+	20020676	20020697
20501286	hsa-miR-151a-5p	8.2	1.39	1.74	3.45	112.19	0.0181	T	T	T	MIMAT0004697	chr8	-	1.42E+08	1.42E+08
20500191	hsa-miR-103a-3p	9.96	3.16	2.21	3.76	111.16	0.0078	T	T	T	MIMAT0000101	chr20	+	3898188	3898210

20500119	hsa-let-7d-5p	9.83	3.23	2.05	3.27	97.29	0.0081	T	T	T	MIMAT0000065	chr9	+	96941123	96941144
20500848	hsa-miR-320a	9.93	3.7	1.27	3.74	74.66	0.01	T	T	T	MIMAT0000510	chr8	-	22102488	22102509
20500746	hsa-miR-140-3p	7.34	1.24	1.21	2.54	68.94	0.0048	T	F	F	MIMAT0004597	chr16	+	69967045	69967065
20500743	hsa-miR-138-5p	7	1.25	2.93	2.23	53.64	0.0261	T	F	F	MIMAT0000430	chr16	+	56892439	56892461
20500121	hsa-let-7e-5p	10.52	5.02	1.2	3.22	45.32	0.0025	T	T	T	MIMAT0000066	chr19	+	52196046	52196067
20503103	hsa-miR-485-5p	6.81	1.31	1.88	2.13	45.03	0.0156	T	T	T	MIMAT0002175	chr14	+	1.02E+08	1.02E+08
20517744	hsa-miR-4284	6.67	1.18	1.93	0.23	44.86	0.0001	T	F	F	MIMAT0016915	chr7	+	73125657	73125674
20500130	hsa-miR-17-5p	6.39	1.14	2.44	0.8	37.97	0.0132	T	F	F	MIMAT0000070	chr13	+	92002872	92002894
20503880	hsa-miR-502-3p	6.88	1.95	1.73	0.94	30.51	0.0001	T	T	T	MIMAT0004775	chrX	+	49779257	49779278
20503876	hsa-miR-500a-3p	6.27	1.41	1.47	0.52	28.99	1.62E-05	T	F	F	MIMAT0002871	chrX	+	49773090	49773111
20504562	hsa-miR-320b	9.56	4.93	1.19	3.58	24.76	0.011	T	T	T	MIMAT0005792	chr1	+	1.17E+08	1.17E+08
20503789	hsa-miR-	5.63	1.02	1.95	2.12	24.53	0.036	T	F	F	MIMAT0002807	chr9	+	20716119	20716140

	491-5p														
20504563	hsa-miR-320c	9.41	5.2	1.43	3.38	18.47	0.0259	T	T	T	MIMAT0005793	chr18	+	19263520	19263539
20501244	hsa-miR-379-5p	5.37	1.32	2.1	0.82	16.55	0.0052	T	F	F	MIMAT0000733	chr14	+	1.01E+08	1.01E+08
20518879	hsa-miR-4485	5.98	2.17	1.13	1.74	13.99	0.0009	T	T	T	MIMAT0019019	chr11	-	10529824	10529843
20501293	hsa-miR-331-3p	4.77	1.05	1.4	1.68	13.16	0.0176	T	F	F	MIMAT0000760	chr12	+	95702256	95702276
20503878	hsa-miR-501-3p	4.29	1.09	1	1.02	9.22	0.004	T	F	F	MIMAT0004774	chrX	+	49774380	49774401
20500194	hsa-miR-106a-5p	4.43	1.33	1.99	0.7	8.57	0.0101	T	F	F	MIMAT0000103	chrX	-	1.33E+08	1.33E+08
20529779	hsa-miR-8069	7.2	8.3	0.75	0.31	-2.14	0.0052	T	T	T	MIMAT0030996	chr21	+	15096563	15096585
20525699	hsa-miR-6869-5p	7.07	8.2	0.29	0.55	-2.18	0.0025	T	T	T	MIMAT0027638	chr20	-	1373584	1373605
20525603	hsa-miR-6821-5p	3.76	5.02	1.05	0.51	-2.4	0.0112	T	T	T	MIMAT0027542	chr22	+	50356514	50356536
20518913	hsa-miR-4516	7.29	8.61	0.69	0.44	-2.5	0.0024	T	T	T	MIMAT0019053	chr16	+	2183121	2183137
20517907	hsa-miR-	6.35	7.69	0.78	0.92	-2.53	0.0346	T	T	T	MIMAT0018076	chr11	+	1.19E+08	1.19E+08

	3656														
20504586	hsa-miR-762	6.61	7.98	0.54	0.31	-2.59	0.0006	T	T	T	MIMAT0010313	chr16	+	30905272	30905293
20515638	hsa-miR-3196	7.53	9.06	0.5	0.34	-2.89	0.0002	T	T	T	MIMAT0015080	chr20	+	61870140	61870157
20522537	hsa-miR-5787	7.32	8.98	0.73	0.77	-3.15	0.031	T	T	T	MIMAT0023252	chr3	+	50264870	50264889
20519636	hsa-miR-4763-3p	4.17	5.96	1.38	1.16	-3.45	0.0495	T	T	T	MIMAT0019913	chr22	+	46509504	46509527
20520198	hsa-miR-5001-5p	2.43	4.39	0.85	1.06	-3.88	0.0425	T	T	T	MIMAT0021021	chr2	-	2.33E+08	2.33E+08

Supplementary Table 6. Details of significantly deregulated miRNA candidates in cytosol in AD vs UC

Transcript ID(Array Design)	AD Cytosol Avg (log2)	HC Cytosol Avg (log2)	AD Cytosol Standard Deviation	HC Cytosol Standard Deviation	Fold Change	P-val	AD Cytosol Expressed	HC Cytosol Expressed	Expressed in Both Conditions	Accession	Chromosome	Strand	Start	Stop
hsa-miR-1915-3p	6.16	8.92	1.68	1.13	-6.79	0.0049	T	T	T	MIMAT0007892	chr10	-	21785505	21785524
hsa-miR-328-5p	4.46	6.94	2	0.46	-5.61	0.0015	T	T	T	MIMAT0026486	chr16	-	67236270	67236292
hsa-miR-7162-3p	1.57	3.78	0.44	2.01	-4.62	0.0222	T	T	T	MIMAT0028235	chr10	-	30657526	30657544
hsa-miR-6749-5p	2.98	5.16	1.57	0.75	-4.53	0.0208	T	T	T	MIMAT0027398	chr11	-	64669901	64669922
hsa-miR-3620-5p	3.85	5.66	1.74	1.17	-3.5	0.0169	T	T	T	MIMAT0022967	chr1	+	2.28E+08	2.28E+08
hsa-miR-638	7.73	9.15	1.63	0.83	-2.66	0.0155	T	T	T	MIMAT0003308	chr19	+	10829095	10829119
hsa-miR-4739	5.89	7.3	1.62	0.93	-2.66	0.0152	T	T	T	MIMAT0019868	chr17	-	77681025	77681049
hsa-miR-4695-5p	3.37	4.66	1.14	0.5	-2.45	0.0179	T	T	T	MIMAT0019788	chr1	-	19209744	19209765
hsa-miR-6727-5p	7.72	8.98	0.72	0.71	-2.41	0.0154	T	T	T	MIMAT0027355	chr1	-	1247919	1247941
hsa-miR-6800-5p	6.92	8.16	0.65	0.47	-2.36	0.0012	T	T	T	MIMAT0027500	chr19	+	50335279	50335299
hsa-miR-6125	8.4	9.6	0.32	0.54	-2.3	0.0029	T	T	T	MIMAT0024598	chr12	+	62654199	62654218
hsa-miR-1202	1.61	2.68	0.27	1.24	-2.1	0.0226	T	T	T	MIMAT0005865	chr6	+	1.56E+08	1.56E+08
hsa-miR-6729-5p	8.72	9.73	0.44	0.66	-2.02	0.003	T	T	T	MIMAT0027359	chr1	+	12089220	12089241

Supplementary Table 7. Details of significantly deregulated miRNA candidates in synaptosomes in AD vs UC

Transcript ID (Array Design)	AD Synaptosome Avg (log2)	HC Synaptosome Avg (log2)	AD Synaptosome Standard Deviation	HC Synaptosome Standard Deviation	Fold Change	P-val	AD Synaptosome Expressed	HC Synaptosome Expressed	Expressed in Both Conditions	Accession	Chromosome	Strand	Start	Stop
hsa-miR-502-3p	7.86	4.65	1.88	0.79	9.23	0.007	T	T	T	MIMAT0004775	chrX	+	49779257	49779278
hsa-miR-500a-3p	6.99	4.87	1.5	1.26	4.33	0.0035	T	T	T	MIMAT0002871	chrX	+	49773090	49773111
hsa-miR-877-5p	5.69	4.34	1.3	0.68	2.55	0.0218	T	T	T	MIMAT0004949	chr6	+	30552109	30552128
hsa-miR-664b-3p	2.63	1.57	1.26	0.39	2.09	0.0386	T	T	T	MIMAT0022272	chrX	+	1.54E+08	1.54E+08
hsa-miR-3196	8.25	9.27	0.52	0.64	-2.04	0.0363	T	T	T	MIMAT0015080	chr20	+	61870140	61870157
hsa-miR-6511b-5p	1.53	2.6	0.54	0.95	-2.09	0.0173	T	T	T	MIMAT0025847	chr16	-	2156721	2156744
hsa-miR-4508	7.17	8.71	1.73	1.1	-2.91	0.0245	T	T	T	MIMAT0019045	chr15	-	23807254	23807270
hsa-miR-1237-5p	5.61	7.34	1.88	0.24	-3.31	0.0231	T	T	T	MIMAT0022946	chr11	+	64136123	64136143
hsa-miR-5001-5p	1.82	3.88	0.72	1.94	-4.16	0.0495	T	T	T	MIMAT0021021	chr2	-	2.33E+08	2.33E+08
hsa-miR-4492	1.63	4.56	0.92	1.62	-7.61	0.0089	F	T	F	MIMAT0019027	chr11	+	1.19E+08	1.19E+08
hsa-miR-4497	1.79	6.05	2.47	0.88	-19.16	0.048	T	T	T	MIMAT0019032	chr12	+	1.1E+08	1.1E+08

Supplementary Data 1. Ingenuity Pathway analysis of synaptosomal and cytosolic miRNAs in UC

Supplementary Data 2. Ingenuity Pathway analysis of synaptosomal and cytosolic miRNAs in AD.

Supplementary Data 3. Ingenuity Pathway analysis of cytosolic miRNAs in AD vs UC.

Supplementary Data 4. Ingenuity Pathway analysis of synaptosomal and cytosolic miRNAs in AD vs UC.
